# Au LSPR Effect Enhanced R‐CeO_2_/G‐C_3_N_4_ S‐scheme Heterojunction for Accelerating CO_2_ Photoreduction Performance

**DOI:** 10.1002/smll.202512107

**Published:** 2026-01-20

**Authors:** Xin Li, Yongsheng Hu, Peng Tian, Yi Lu, Qiong Wu, Binrong Li, Maobin Wei, Xiaofei Yang, Lili Yang, Huilian Liu, Alberto Vomiero

**Affiliations:** ^1^ Key Laboratory of Functional Materials Physics and Chemistry (Ministry of Education) Jilin Normal University Changchun P. R. China; ^2^ National Demonstration Center For Experimental Physics Education Jilin Normal University Siping P. R. China; ^3^ International Innovation Center For Forest Chemicals and Materials College of Science Nanjing Forestry University Nanjing P. R. China; ^4^ National and Local Joint Engineering Laboratory of Municipal Sewage Resource Utilization Technology School of Environmental Science and Engineering Suzhou University of Science and Technology Suzhou P. R. China; ^5^ Division of Materials Science Department of Engineering Sciences and Mathematics Luleå University of Technology Luleå Sweden; ^6^ Department of Molecular Sciences and Nanosystems Ca’ Foscari University of Venice Venezia Italy

**Keywords:** CeO_2_, CO_2_ photoreduction, g‐C_3_N_4_, LSPR, S‐scheme

## Abstract

Excellent CO_2_ adsorption ability and fast photogenerated carriers’ supply are vital conditions for efficient CO_2_ photoreduction. In this paper, Au localized surface plasmon resonance (LSPR) has been successfully applied in a R‐CeO_2_/g‐C_3_N_4_ S‐scheme heterojunction photocatalyst for CO_2_ photoreduction. R‐CeO_2_/Au/g‐C_3_N_4_ (CAC‐2) exhibited excellent CO_2_ photoreduction performance and great stability. The CO yield over CAC‐2 is about 50.58 µmol·g^−1^·h^−1^ under UV–vis light irradiation, which is about 6.7 and 6.0 times higher than that of R‐CeO_2_ and g‐C_3_N_4_, respectively. FDTD simulation, DFT calculation and photoelectrochemical tests together prove the introduction of Au NPs not only enhances the photogenerated carriers’ separation efficiency, but also decreases the formation energy barrier of the important intermediate *COOH, which is beneficial for the CO_2_ photoreduction to CO. N_2_/CO_2_ adsorption‐desorption curves indicated that the CAC‐2 ternary composite had the largest specific surface area and the best CO_2_ adsorption capacity. Meanwhile, DFT calculation confirmed that the reduction sites of the CAC‐2 had the highest electron density, which can synergistically enhance the CO_2_ photoreduction activity. The improvement of photocatalytic performance can be attributed to the synergistic enhancement of Au LSPR effect and S‐scheme heterojunction at the interface. Based on the in situ FTIR, in situ ESR, and ^13^C isotope tracer experiment, a potential LSPR effect‐enhanced S‐scheme heterojunction catalytic mechanism has been provided, which may represent a significant advancement in the field.

## Graphical abstract

This study develops an Au localized surface plasmon resonance (LSPR) enhanced S‐scheme heterojunction between oxygen vacancy‐rich CeO2 (R‐CeO2) and graphitic carbon nitride (g‐C3N4) for efficient CO2 photoreduction. The ternary catalyst achieves high CO yield by synergistically improving carrier separation and reducing energy barriers, as validated through experiments and simulations.

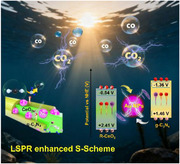

## 1 Introduction

In recent years, photocatalytic CO_2_ reduction technology, driven by inexhaustible and environmentally friendly solar‐energy, has aroused extensive research enthusiasm among researchers, and is viewed as the ideal CO_2_ resource utilization technology [1, 2, 3]. In general, photocatalytic process includes these steps: (1) The adhesion of CO_2_ molecules on the photocatalyst; (2) Generation of electron‐hole pairs with strong redox performance inside the photocatalyst under light irradiation; (3) Photogenerated carriers transfer to the catalysts’ surface; (4) Reaction of the photogenerated carriers with strong redox ability with CO_2_ and H_2_O molecules to realize the conversion of CO_2_ into other C‐based products [4, 5, 6]. Therefore, the photogeneration & transmission behaviors of carriers are the decisive factors for the photocatalytic ability of the catalyst. So, the construction of catalytic materials with high photo‐absorption performance, outstanding CO_2_ adsorption performance, excellent redox ability, and photogenerated carriers’ transmission ability is an important prerequisite for highly efficient CO_2_ photocatalytic reaction.

In recent years, S‐scheme heterojunctions have been proposed as a promising solution for constructing efficient CO_2_ photoreduction materials due to their ability to maximize the retention of photogenerated carriers for redox reactions and the effective improvement of electron‐hole pairs’ separation performance [7]. Cerium oxide (CeO_2_), with great stability and Ce^4+^→Ce^3+^ valence change capacity, has been widely applied in photocatalysis. From the viewpoint of electronic band‐structure, the positive valence band level (*E_VB_* ≈ +2.0 V) makes CeO_2_ have strong oxidation ability under light excitation, which shows that it is a good oxidizing semiconductor for constructing the S‐scheme heterojunction materials [8]. Graphitic carbon nitride (g‐C_3_N_4_) with enough negative conduction band potential (*E_CB_* ≈ −1.0 V) and great material stability has been strongly researched in the photocatalytic CO_2_ reduction field [9]. However, the swift reassociation of carriers photogenerated in g‐C_3_N_4_ is a reason for its low photocatalytic activity, so the CO_2_ photoreduction efficiency of the g‐C_3_N_4_ is not very satisfactory [10]. In our earlier study, we synthesized the 2D/1D/2D rGO/R‐CeO_2_/g‐C_3_N_4_ S‐scheme catalyst for CO_2_ photoreduction and studied the carrier migration process in the ternary composite [11]. The multidimensional electron transfer process at the interface was the key reason for the superior CO_2_ photoreduction ability. However, the carrier's separation velocity at the 1D/2D S‐scheme interface cannot meet the demands of the CO_2_ photoreduction reaction. The construction of the S‐scheme heterojunction still needs further design and optimization.

Recent researches prove that the modification of S‐scheme catalyst by using metals with strong local surface plasma resonance effect (LSRP) can provide a large number of photothermal electrons to improve photocatalytic activity while improving photogenerated carrier generation and transfer performance inside the heterojunction, and further improve the photoreduction activity of the heterojunction catalysts [12, 13]. For example, Shen et al. prepared a Cu_2_WS_4_/MoS_2_‐Au plasmonic S‐scheme heterojunction for the efficient Cr^6+^ reduction and a Benzophenone‐1 oxidation under visible‐light irradiation. The Au nanoparticles (NPs) with LSPR effect further enhanced the transfer performance of electron and hole pairs while improving the electron‐hole pairs migration efficiency in Cu_2_WS_4_/MoS_2_ S‐scheme heterostructure, thereby obtaining excellent photocatalytic activity [14]. In addition, we have previously reported Au superior photoreduction efficiency in ZnO/g‐C_3_N_4_ heterojunction for efficient CO_2_ photoreduction, also showing that the modification of Au NPs can greatly enhance the transmission of carriers inside the composite [15]. Therefore, the introduction of Au NPs with good visible light plasmonic absorption ability in an S‐scheme heterojunction composite is a potential method to further improve its CO_2_ photoreduction activity.

In this research, we selected Au NPs as the plasmonic excitation source, 1D oxygen vacancy (O_Vs_)‐rich CeO_2_ nano‐rods (R‐CeO_2_) as the oxidation catalyst, and g‐C_3_N_4_ as the reduction catalyst to construct the LSPR effect‐enhanced core‐shell S‐scheme heterojunction for CO_2_ photoreduction. The process of carriers’ generation, transfer, and separation was investigated by combining experiments, physical simulations, and density functional theory (DFT) calculations, and a possible photocatalytic enhancement mechanism was discussed.

## 2 Results and Discussion

### 2.1 Crystal‐Phase Structure and Elemental Composition Analysis

**FIGURE 1:**
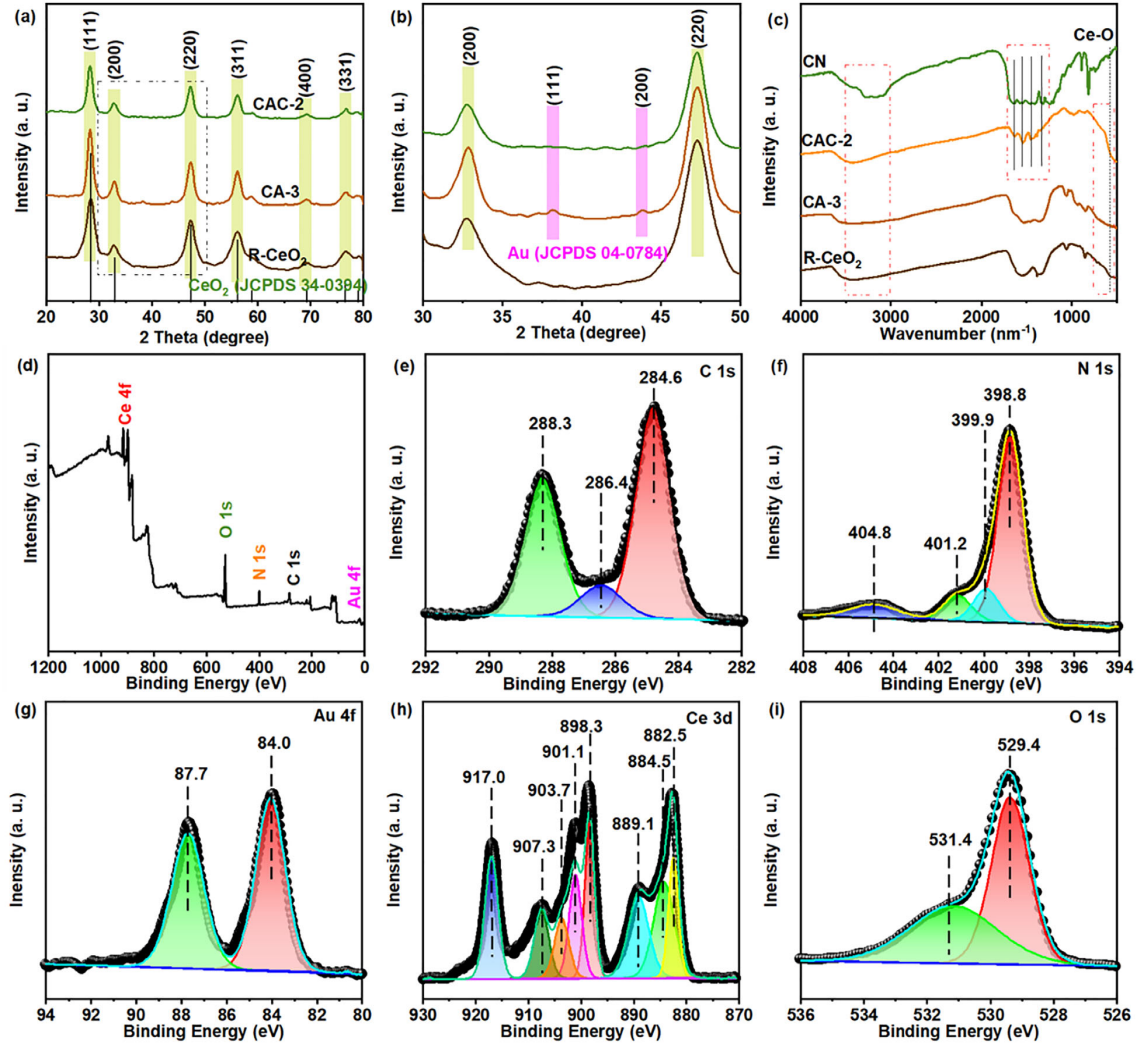
XRD patterns of prepared R‐CeO_2_, CA‐3, and CAC‐2 in 20°–80°(a), and 30°–50° (b); FTIR spectra of g‐C_3_N_4_, R‐CeO_2_, CA‐3, and CAC‐2 (c); XPS spectra of the CAC‐2 composite photocatalyst: survey scan (d), C 1s (e), N 1s (f), Au 4f (g), Ce 3d (h), and O 1s (i), respectively.

Powder X‐ray diffraction (XRD), Fourier transform infrared spectroscopy (FTIR), and X‐ray photoelectron spectroscopy (XPS) were applied to investigate the phase‐structure, surface characteristics, chemical bonds, and elemental composition of the samples. As shown in Figure 1a, six peaks at around 28.5°, 32.9°, 47.3°, 56.2°, 69.3°, and 76.5° appeared in the XRD patterns of all the catalysts, which can be attributed to the (111), (200), (220), (311), (400), and (331) crystal faces of CeO_2_ (PDS#34‐0394) [16]. In Figure 1b, the presence of two weak signals can be detected at about 38.2° and 44.3° in the XRD pattern of CA‐3 in the enlarged view of Figure 1a, which can be indexed as the characteristic (100) and (200) diffraction peaks of Au (JCPDs 04–0784) [17]. When g‐C_3_N_4_ was covered on the surface of CA‐3, the diffraction peak of the ternary composite did not change significantly, except for a small attenuation, which may be caused by the small load of g‐C_3_N_4_. From the above analysis of the characteristic peaks in CAC‐2 ternary photocatalyst, we can preliminarily speculate that the compound is composed of CeO_2_ and small Au crystals (not clearly confirmed from XRD), while the presence of g‐C_3_N_4_ still needs further exploration. Figure 1c displays the FTIR spectra of R‐CeO_2_, CA‐3, CAC‐2, and pure g‐C_3_N_4_. The extremely weak vibration of the Ce─O bond in R‐CeO_2_, CA‐3, and CAC‐2 can be found at about 578 cm^−1^ [18]. The typical FTIR fingerprint peaks of pure g‐C_3_N_4_, asymmetric stretching vibration of C‐N‐C in the aromatic ring (1258 cm^−1^), C‐N symmetrical stretching vibration (1339 cm^−1^), C═N stretching vibration (1548 cm^−1^), the aromatic ring skeleton vibrates (1637 cm^−1^), can be detected between 1100–1700 cm^−1^ in FTIR curves of pure g‐C_3_N_4_ and CAC‐2 [19]. These characteristic peaks could not be observed in the CA‐3 and R‐CeO_2_ samples, providing further evidence for the successful coating of g‐C_3_N_4_ on R‐CeO_2_. The broader peaks at around 3000–3700 cm^−1^ are caused by the H_2_O and amino‐group on the surface of all samples [20].

The XPS survey spectrum in Figure 1d illustrates the existence of C, N, O, Ce, and Au elements in the CAC‐2 composite. As shown in Figure 1e (C 1s), the binding energy peaks at approximately 284.6, 286.4, and 288.3 eV relate to the characteristic peaks of C─C, C─O, and C═O groups in the ternary composite, respectively [21]. Figure 1f displays the N 1s XPS spectrum of the composite, and four clear peaks can be fitted at about 398.8, 399.9, 401.2, and 404.8 eV, which correspond to the typical peaks of C─N═C, N─(C)_3_, C─NH_2_ groups, and π‐excitation of g‐C_3_N_4_ [22]. As shown in Figure 1g, the typical peaks of Au can be found at about 84.0 and 87.7 eV, respectively [22]. In Figure 1h, the Ce 3d XPS spectrum can be analyzed using eight split peaks. The binding‐energy peaks at approximately 882.5, 884.5, 889.1, and 898.3 eV confirm the presence of Ce^3+^ state, while the peaks at 901.1, 903.7, 907.3, and 917.0 eV are attributed to the Ce^4+^ state in the catalyst. Figure 1i exhibits the corresponding O 1s XPS curve. The binding energy peaks at approximately 529.4 eV and 531.4 eV can be indexed with the Ce─O band, and the adsorbed oxygen on oxygen vacancy [23]. Besides, the XPS peak fitting information of Ce ions in CAC‐2 is shown in Table S1. And the Ce^3+^/Ce^4+^ ratio in this sample can be calculated as about 1.33, which proves the presence of a high concentration of oxygen defects in CeO_2_ [24]. Electron paramagnetic resonance (EPR) was applied to further research the surface defect concentration of materials, and the corresponding curves are exhibited in Figure S1. The typical signal from O_Vs_ defect is present at the g‐value of about 1.96 in all samples [25]. The abundant O_Vs_ structure is conducive to the coupling of Au NPs and g‐C_3_N_4_ on R‐CeO_2_ surface. The O_Vs_ defect signal intensity of binary and ternary composites is obviously reduced compared to the pure R‐CeO_2_, which further verifies the successful preparation of core‐shell composite materials [26]. The above XRD, FTIR, and XPS analysis prove that the CAC‐2 is composed of the CeO_2_, Au and g‐C_3_N_4_.

### 2.2 Morphological and Structural Analysis

**FIGURE 2:**
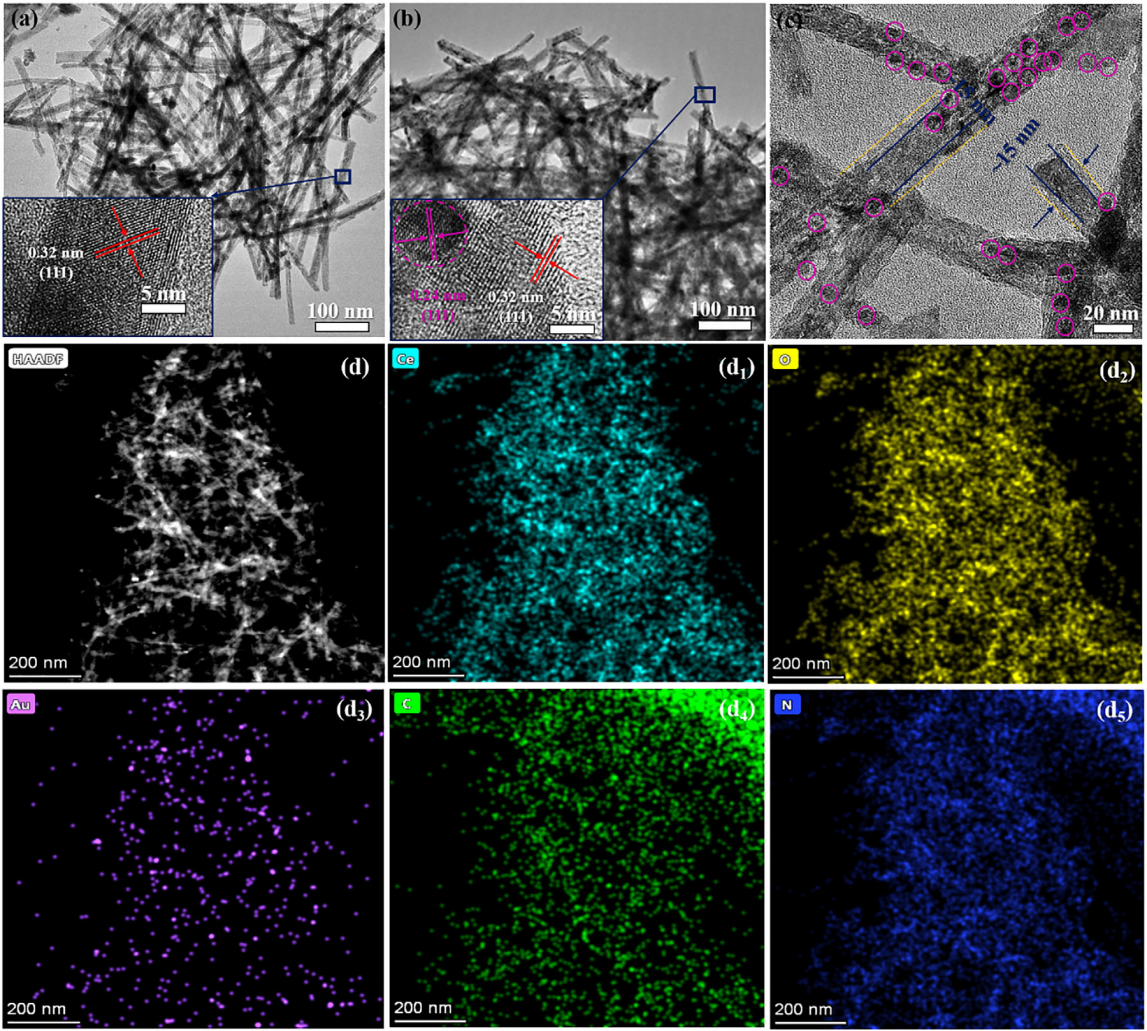
TEM images of pure R‐CeO_2_ (a), CA‐3 (b), and CAC‐2 (c); HAADF‐STEM image of CAC‐2 (d), and the corresponding elemental mapping results of it (d1‐d5).

The morphologies and chemical composition of the obtained catalysts were researched by transmission electron microscopy (TEM), high‐resolution transmission electron microscopy (HR‐TEM), and EDS mapping. Figure 2a–c shows the TEM images of R‐CeO_2_, CA‐3, and CAC‐2, respectively. The prepared CeO_2_ has a regular nano‐rod structure with a diameter of ≈20 nm, and a length of ≈500 nm, and the nano‐rods’ surface is relatively smooth. The inset of Figure 2a exhibits the HR‐TEM of R‐CeO_2_ with a clear interplanar spacing of approximately 0.32 nm, which can be attributed to the (111) lattice plane of CeO_2_ [27]. There are some nano‐particles with a diameter of 5 nm adsorbed on R‐CeO_2_ after the UV‐reduction process in Figure 2b [28]. Inset in Figure 2b displays the HR‐TEM image of CA‐2, and the crystal plane spacings of 0.32 and 0.24 nm can be related to the (111) lattice planes and (111) of CeO_2_ and Au, respectively [29]. After the typical impregnation‐calcination process, a rough shell structure with a thickness of about 5 nm appeared on the surface of CA‐3 in Figure 2c.

To further confirm the elemental composition and distribution of CAC‐2 composite, EDS mapping was used, and the EDS energy spectrum, specific mapping‐images, and the content of each element in CAC‐2 are shown in Figure S2, Figure 2d (d‐d5), and Table S2, respectively [30]. The results of Figure S2 prove the presence of each element in the ternary composite. Figure 2d is the HAADF‐STEM image of the selected area of CAC‐2. Many nanoparticles and shaped shells covered around the smooth surface of nanorods are present. Figure 2d1–d5 shows the Ce, O, Au, C, and N elemental mapping distribution of CAC‐2, respectively. CAC‐2 is a framework composed of Ce and O elements, indicating that the main body of the ternary composite is R‐CeO_2_. Au is well dispersed in the form of granular particles on the composite's surface, reflecting the uniform distribution of Au NPs [31]. The slight inconsistency between the distribution of C element and the morphology of CAC‐2 is caused by the ultra‐thin carbon film from the TEM grid. However, the loose distribution of the N element and the presence of each element in CAC‐2, compared to the Ce and O elements, further prove the successful coverage of the g‐C_3_N_4_ shell. Based on the EDS and mapping results, Table S2 summarizes the proportions of each element in the composite. Among them, the proportion of Au is over 3.5 wt.%. The Au NPs sizes of CAC‐2 displayed in TEM (in Figure S3a) were statistically analyzed. The specific results are shown in Figure S3b, which indicates an average size of Au NPs equal to …±… nm [32]. Finally, the TEM, HR‐TEM, and XPS results unequivocally demonstrate the successful preparation of Au NPs modified R‐CeO_2_@g‐C_3_N_4_ composite.

### 2.3 Band Gap Structure Analysis

**FIGURE 3:**
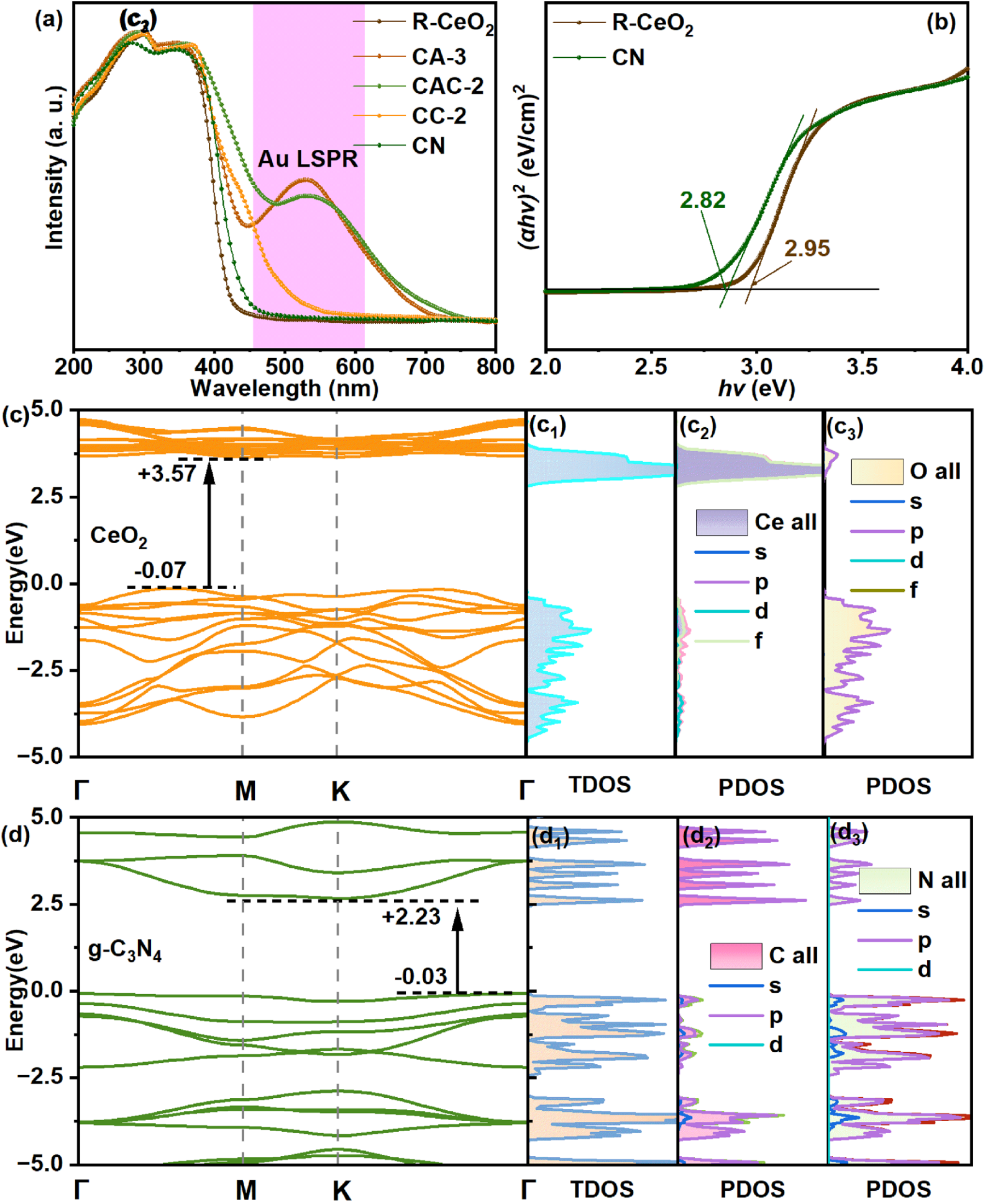
UV–vis DRS absorption spectra (a). Tauc plots for the calculation of the band gaps (b). DOS results of pure CeO_2_ (c) and g‐C_3_N_4_ (d).

The enhanced photo‐absorption is of significance to enhance the CO_2_ photocatalytic reduction performance of the catalysts. The UV–vis diffuse reflection spectra (UV–vis DRS) of R‐CeO_2_, CA‐3, CAC‐2, and CC‐2 composites are depicted in Figure 3a. It is evident that R‐CeO_2_ and g‐C_3_N_4_ exhibit similar light adsorption properties, with an adsorption edge at about 420 and 432 nm, respectively. After the incorporation of Au NPs, a characteristic LSPR peak appears at approximately 550 nm, and the light adsorption range is further extended to about 700 nm [33]. After the coating of g‐C_3_N_4_ shell (sample CAC‐2), the plasmonic absorption is still present and very strong, testifying that the external g‐C_3_N_4_ layer does not negatively affect the ability of gold nanoparticles to exert plasmonic absorption. Compared to the binary g‐C_3_N_4_/CeO_2_ composite, the incorporation of Au NPs can notably improve the photo‐absorption capability of the heterojunction. which is helpful for the photocatalytic CO_2_ reduction [34]. The band gap (*E_g_*) results of R‐CeO_2_ and g‐C_3_N_4_ exhibited in Figure 3b are estimated to be about 2.95 and 2.82 eV, respectively.

Furthermore, the density of states (DOS) calculation by Heyd‐Scuseria‐Ernzerhof (HSE06) functional method was used to analyze the specific band gap structures of prepared R‐CeO_2_, and g‐C_3_N_4_samples, and the specific band gap structures are displayed in Figure 3c,d, respectively. The bandgap widths of pure R‐CeO_2_ and g‐C_3_N_4_ were calculated as 3.64 and 2.26 eV, respectively (VS. NHE) [35]. The gap width in the theory of them is slightly different from the experimental results, which might be due to the differences between the theoretical model and the macroscopic materials involved in the experiment [28, 29, 36]. Partial density of states (P‐DOS) results of CeO_2_ in Figure 3c1–c3 display that Ce 4f and O 2p orbitals give the main contribution to the CB and VB of R‐CeO_2_, respectively. Figure 3d1–d3 exhibit that C 2p and N 2p orbitals provide the VB and CB of P‐DOS in g‐C_3_N_4_ [37]. The above results indicate that the Ce and N sites in the composite are potential reduction sites, while the O and C sites are potential oxidation sites.

#### 2.3.1 CO_2_ Photoreduction Performance Analysis

**FIGURE 4:**
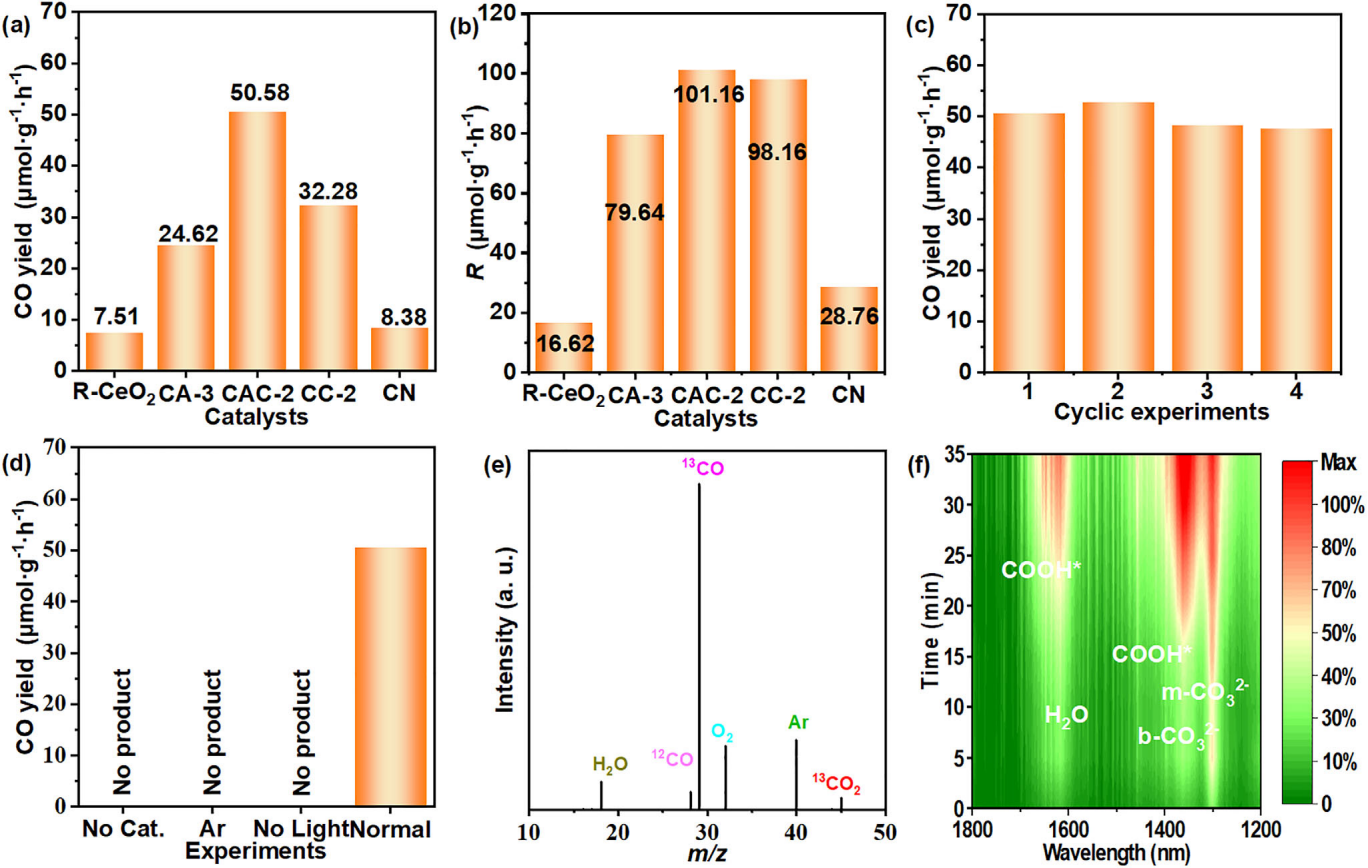
CO yield (a) and electron utilization (b) of R‐CeO_2_, CA‐3, CAC‐2, CC‐2 and CN; cyclic experiments with CAC‐2 as the catalyst (c); CO_2_ photoreduction experiments under the different conditions (d); 13CO_2_ isotope labeling photoreduction experiment (e) and in situ FTIR spectra of CO_2_ photoreduction with CAC‐2 as the catalyst (f).

To test the CO_2_ photoreduction of all catalysts, the specific CO_2_ photoreduction system in Figure S4 has been applied, and the CO_2_ photoreduction ability of the obtained catalysts is shown in Table S3 and Figure 4a. The photocatalytic CO_2_ reduction performance of all samples was tested under irradiation of a Xe‐lamp for 4 h [38]. The corresponding Xe‐lamp emission spectrum is shown in Figure S5. In our study, CO was detected as the main gas product, while very small amounts of CH_4_ as a by‐product can be detected in this reaction system. The CO_2_ photoreduction performance and CO selectivity of all prepared catalysts aredisplayed in Table S3. It is clear that the photocatalytic ability of all composite catalysts (R‐CeO_2_/g‐C_3_N_4_ and R‐CeO_2_/Au/g‐C_3_N_4_) is significantly enhanced compared with the R‐CeO_2_ and g‐C_3_N_4_. As the Table S3, the CO_2_ photoreduction ability of all CC binary and CAC ternary samples is first‐increased and then decreased, which may be caused by the excessive Au NPs or g‐C_3_N_4_ affecting the transfer ability of the photogenerated carriers between the composites.

Figure 4a shows the yield of CO with the R‐CeO_2_, g‐C_3_N_4_, CA‐3, and CAC‐2 as the catalyst, respectively. The ternary CAC‐2 composite exhibits the best photocatalytic performance, and the selectivity of CO is as high as 100%. The CO yield over CAC‐2 is about 50.58 µmol·g^−1^·h^−1^ under irradiation of the UV–vis light, which is about 6.7 and 6.0 times higher than that one of R‐CeO_2_ (7.51 µmol·g^−1^·h^−1^) and g‐C_3_N_4_ (8.38 µmol·g^−1^·h^−1^), respectively. To further verify the contribution of the modification of Au NPs to the photocatalytic performance, the CO_2_ photoreduction efficiency of the CC‐2 and CAC‐2 samples was tested under the irradiation of visible light (>420 nm). The corresponding CO yield with the CC‐2 and CAC‐2 as the catalyst in Figure S6 is about 3.28 and 18.87 µmol·g^−1^·h^−1^, which proves that the LSPR effect of Au NPs can greatly improve the photocatalytic performance of the composite [39].

In CO_2_ photocatalytic reduction, the utilization efficiency of electron flux of the sample (*R*) was calculated as in our previous report [40]. Figure 4b displays the *R* of catalysts in photocatalytic CO_2_ reduction (calculated as Equation S1). It proves that CAC‐2 exhibits the greatest utilization efficiency of photogenerated electrons. The specific values further prove that the CAC‐2 has the best photocatalytic CO_2_ reduction ability compared to the other catalysts. In general, the samples’ catalytic stability is a vital aspect in the process of CO_2_ photoreduction [41]. Four successive photocatalytic CO_2_ reduction cyclic reactions were taken to analyze the catalytic stability of CAC‐2, with the reaction results presented in Figure 4c. Following each cycle of the CO_2_ photoreduction reaction, the reaction system was cleaned, and the sample was required to be degassed. The C 1s and N 1s XPS spectra of CAC‐2 before and after the reaction, shown in Figure S7, are not significantly changed, which proves that the CAC‐2 exhibits great photocatalytic performance and excellent material stability. To verify the origin of the C‐element in photogenerated CO molecules, the reduction process of CO_2_ emissions was investigated under various conditions. In Figure 4d, in the absence of light, sample, or CO_2_, no CO‐gas could be obtained. In addition, the ^13^C isotope‐calibration reaction was carried out under the same photoreduction conditions, except that a mixture of ^13^CO_2_/Ar gas (*v/v*% = 1:2) was used to replace the ^12^CO_2_ gas. The corresponding result is displayed in Figure 4e: the steep peak with *m*/*z* value of about 29 is caused by the presence of ^13^CO. The much weaker *m*/*z* peak at about 28 corresponds to ^12^CO, which is caused by the mixing of air into the reaction device. The detected peaks with *m*/*z* values of about 45, 40, 32, 18, and 4 can be identified as the unreduced ^13^CO_2_, the filled Ar, the formed O_2_ and H_2_O, and the instrument carrier gas (He), respectively. The O_2_ present in the mass spectrum indirectly proves the occurrence of the overall CO_2_ photoreduction reaction without any sacrificial agent. As a complete redox reaction, the reduction and oxidation performance of the CAC‐2 catalyst was analyzed by a more advanced gas‐chromatography which can detect the O_2_. The specific CO and O_2_ yields are shown in Figure S8 and Table S4. The CO production rate is similar to the previous measurement (53.00 µmol·g^−1^·h^−1^), while the O_2_ yield is approximately 20.57 µmol·g^−1^·h^−1^. These results further indicate that the CAC‐2 ternary composite possesses excellent catalytic ability for the overall CO_2_ photoreduction reaction. The results we mentioned above further indicate that the C‐element in product (CO) is derived from the CO_2_ photoreduction rather than the breakdown of the materials in this reaction system [42, 43].

The CO_2_ adsorption process and photoreduction reaction with the CAC‐2 as the catalyst were monitored in real time by the in situ FTIR spectroscopy, and the corresponding curves and 2D map are displayed in Figure S9 and Figure 4f, respectively. The peaks at about 2300 and 2400 cm^−1^ in Figure S6 are caused by the *CO_2_, where “*” means the specific active‐site catalyst's surface [44]. The CO_2_ adsorption peak intensity of CAC‐2 increases with the dark reaction time (14 min), and it reached saturation after 14 min. Figure 4f displays the in situ FTIR characteristic peaks on the surface of CAC‐2 in 1200–1800 cm^−1^ after the photo‐reaction (35 min). Multiple carbonates and bicarbonates can be found in these processes, which are caused by the reaction of H_2_O and CO_2_. The obvious peaks appearing at 1305 and 1362 cm^−1^ can be attributed to the bi‐dentate (b‐CO_3_^2−^) and mono‐dentate (m‐CO_3_^2−^) carboxylate species [41], which proves the presence of CO_3_^2−^ group. The FTIR peaks appearing at about 1462 and 1669 cm^−1^ correspond to COOH*, a main intermediate for the conversion of CO_2_ to CO [45, 46]. Besides, the levels of these substances rise steadily as the irradiation time increases, providing additional evidence that the CO originated from the CO_2_ photoreduction process.

### 2.4 Photo‐Electrochemical (PEC) Analysis

**FIGURE 5:**
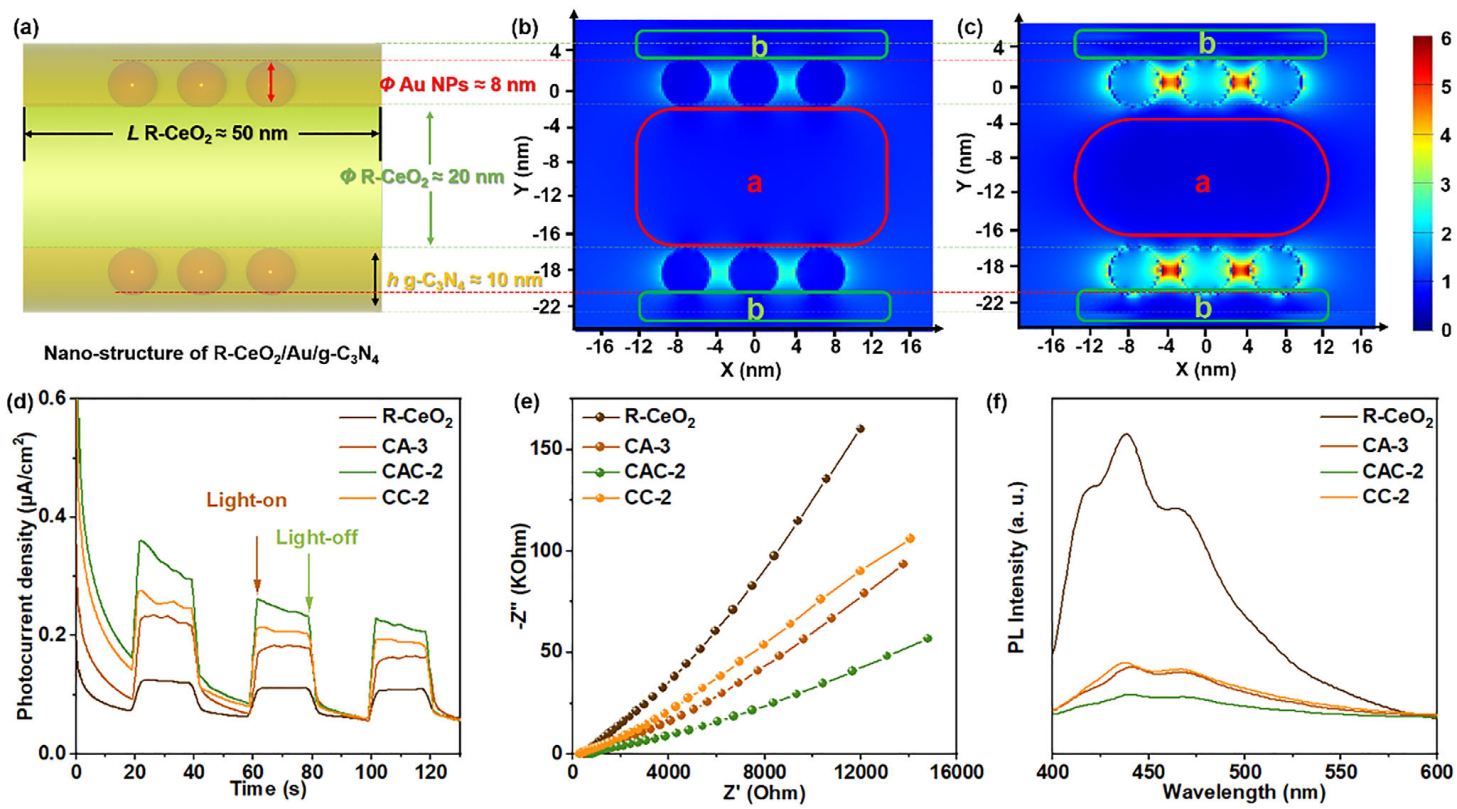
Nano‐structure of CAC‐2 (a); FDTD simulation result of CAC‐2 at 360 nm excitation (b) and 550 nm (c); TPR (d), EIS (e), and PL spectra (f) of prepared samples, respectively.

The ratio of photogenerated carrier separation is a crucial factor that influences photocatalytic performance. In general, a consistently high carrier's separation ratio is a clear indicator of the great photocatalytic ability of the catalyst [47]. The finite‐difference time‐domain simulation (FDTD) and PEC tests were run to compare the separation performance of carriers within the CAC‐2. Based on the UV–vis DRS results, we selected 360 nm (where all semiconductors can be excited) and 550 nm (where only Au can be excited) as the excitation wavelengths to simulate the localized electromagnetic field (LEF) intensity of CN/CeO_2_ and Au under the excitation conditions, respectively. The model of this ternary catalyst (CeO_2_‐Au‐g‐C_3_N_4_, *Φ*(R‐CeO_2_) ≈20 nm, *Φ*(Au NPs) ≈8 nm, *h*(g‐C_3_N_4_) ≈10 nm) is simulated in Figure 5a and Figure S10, which is constructed by the TEM results in Figure 2c. The corresponding FDTD simulated 2D mapping results in Figure 5b,c show that the LEF is mainly concentrated around Au NPs in the g‐C_3_N_4_ coating layer covered on the surface of CeO_2_ when excited by 360 and 550 nm, and there is also a certain degree of radiation to the CeO_2_/g‐C_3_N_4_ interface [48]. Still, the LEF enhancement intensity of the interface area of CeO_2_ (part a) and the outer edge of g‐C_3_N_4_ (part b) inside Figure 5c is much lower than that of the corresponding parts of Figure 5b, which may be because of the 550 nm light wavelength cannot be absorbed by CeO_2_ or g‐C_3_N_4_. Thus, the LEF of CeO_2_ NRs’ surface or g‐C_3_N_4_ is caused by the Au NPs’ LSPR effect under the 550 nm light excitation. Besides, the LEF of the interfaces of the composite excited by the 550 nm light is greatly stronger than that excited by the 360 nm light, which demonstrates Au NPs LSPR effect with sufficient capability to speed up the transmission processes of carriers within CeO_2_ and g‐C_3_N_4_ [49, 50].

The transient photocurrent response (TPR) results of all prepared catalysts further confirm this hypothesis. As Figure 5d shows, the CeO_2_/g‐C_3_N_4_ has a higher TPR signal than each single component sample, and the signal from the CAC‐2 ternary composite is significantly stronger than the CeO_2_/g‐C_3_N_4_, which may be due to the Au NPs’ LSPR effect at interfaces of the binary interface [51]. The arc radius of the Nyquist plot of the CAC‐2 in the EIS curves of Figure 5e is much smaller than that of all the other samples, which confirms that the electron transport ability of CC‐2 can be further improved by the introduction of Au NPs. Figure 5f displays the PL results of all the samples. The CAC‐2 ternary composite exhibits the lowest PL intensity, which confirms that the radiative recombination of photogenerated carriers inside CAC‐2 is effectively inhibited [52]. The high efficiency of photo‐electric conversion and quick separation efficiency of electron‐hole pairs photogenerated in the catalyst, which are present in the CAC‐2 sample, are essential prerequisites for an outstanding photocatalyst.

### 2.5 Charge Transfer Path Analysis

**FIGURE 6:**
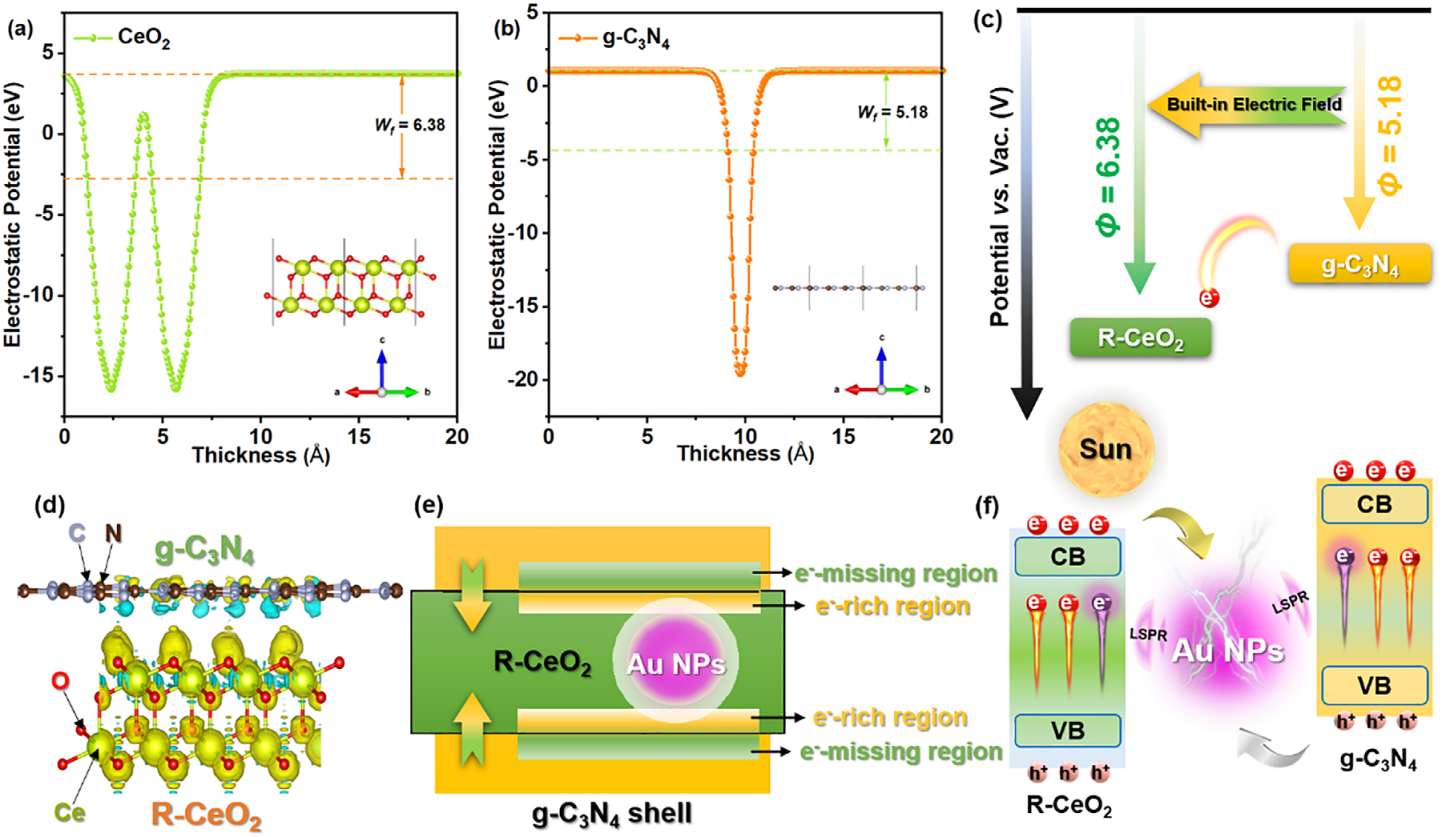
Calculated work‐function results of R‐CeO_2_ (a) and g‐C_3_N_4_ (b); the formation of a built‐in electric field at binary composite (c); DCD results of R‐CeO_2_/Au/g‐C_3_N_4_ (d); scheme of electron distribution results at the interfaces of CAC‐2 (e); and the transfer path of photogenerated carriers at the CAC‐2 interfaces (f).

To substantiate the charge transmission and separation paths in the CeO_2_/Au/g‐C_3_N_4_ ternary sample, the work‐function values (*Φ*) of CeO_2_ and g‐C_3_N_4_ were calculated by the DFT method, and the corresponding model structures (double/single‐layer crystal planes) and results are shown in Figure 6a,b. The specific *Φ* of CeO_2_ and g‐C_3_N_4_ is about 6.38 and 5.18 eV, which proves that CeO_2_ processes a lower Fermi level (*E_f_*) than the g‐C_3_N_4_, promoting the transmission of material electrons in the interface from g‐C_3_N_4_ to CeO_2_ (Figure 6c) [53].

To characterize this phenomenon more intuitively, we calculated the differential charge density between the g‐C_3_N_4_/CeO_2_ interface, and the corresponding image is displayed in Figure 6d. In this image, the cyan and yellow areas at the interface correspond to the electron missing‐region and the electron‐rich region, respectively. The cyan and yellow areas mainly focused on the side of g‐C_3_N_4_ and the side of CeO_2_, respectively. The averaged charge density (*Δρ*) as a function of the vertical axis, integrating all the cyan and yellow regions, is shown in Figure S11. The *Δρ* on the g‐C_3_N_4_ side is negative, while that on the CeO_2_ side is positive, indicating that an e^−^‐missing region has formed on g‐C_3_N_4_’s surface, and an e^−^‐rich region has formed at the CeO_2_ surface, respectively (Figure 6e). The XPS comparison results in Figure S12 show that the Ce atom gains electrons while the N atom loses electrons after forming the composite, which is consistent with the DFT results above. Therefore, the direction of the built‐in electric field (IEF) is from g‐C_3_N_4_ to CeO_2_, which is caused by the difference in charge density between these two materials [54]. Under UV–vis light excitation, electrons can be promoted from VB to CB within g‐C_3_N_4_ and CeO_2_. Subsequently, driven by the IEF, electrons in the conduction band (CB) of CeO_2_ migrate to recombine with holes in the valence band (VB) of g‐C_3_N_4_. This process leads to the spatial separation and accumulation of electrons on g‐C_3_N_4_ and holes on CeO_2_ [55]. This phenomenon has led to the g‐C_3_N_4_ within the composite becoming the primary site for CO_2_ photoreduction. The carrier generation and transfer processes can be further enhanced by the LSPR effect of Au NPs (Figure 6f) [56]. Based on the PEC and DFT results, it can be proved that the photogenerated charge transfer path of the ternary composite satisfies the S‐scheme charge separation path. Therefore, the synergistic effect of IEF and the LSPR of Au NPs efficiently maximizes the separation ratio of the photogenerated carriers in CAC‐2, thus improving the CO_2_ photoreduction performance.

### 2.6 CO_2_ Photoreduction Mechanism Analysis

**FIGURE 7:**
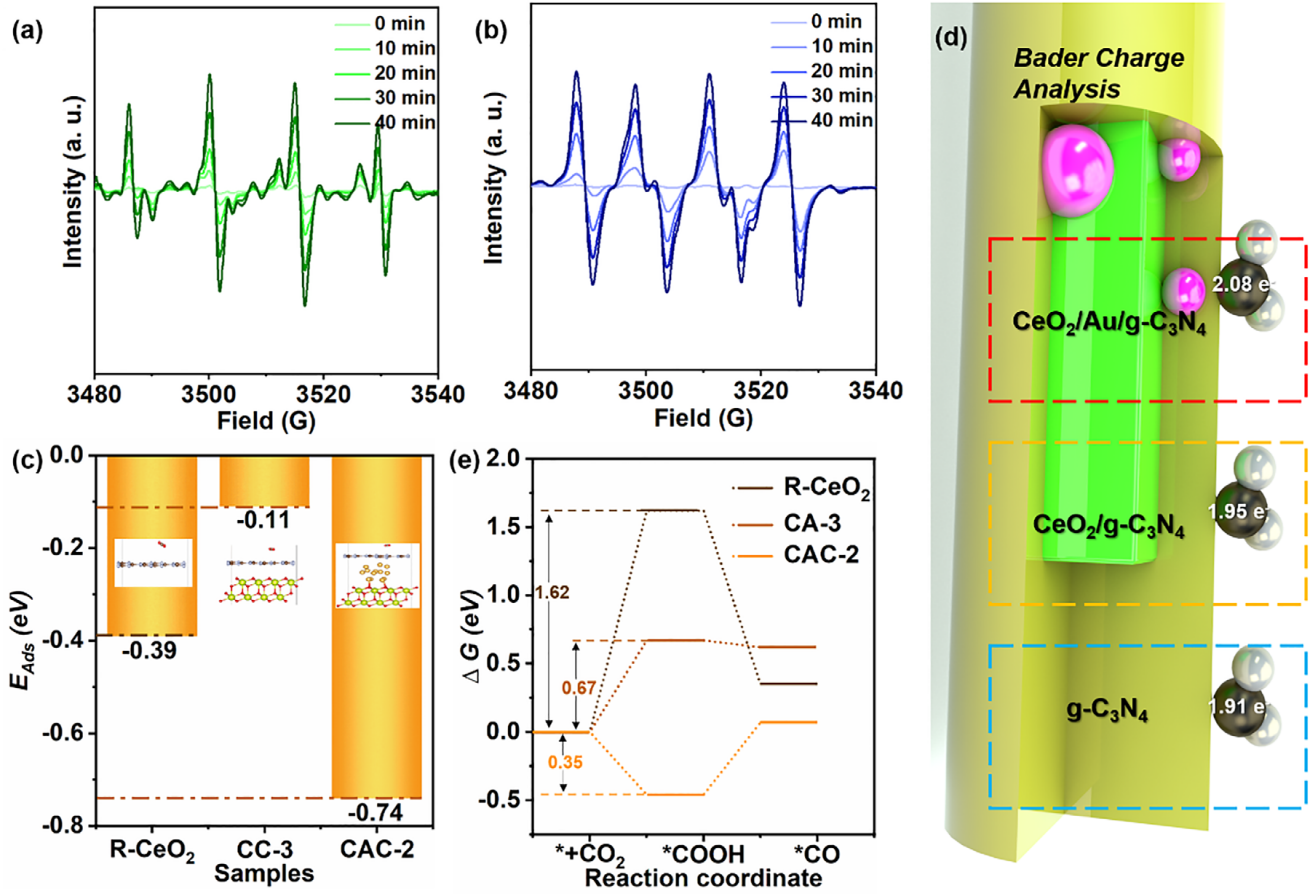
In situ DMPO‐O_2_^−^ and DMPO‐OH radicals’ curves of CAC‐2 (a,b); *E_Ads_*, Bader charge analysis, and Gibbs Free‐energy of CeO_2_, CeO_2_/g‐C_3_N_4_, and CeO_2_/Au/g‐C_3_N_4_(c‐e).

The band gap structure of prepared R‐CeO_2_ and g‐C_3_N_4_ was investigated by the ultraviolet photoelectron spectroscopy (UPS) spectra (Figure S13a,b), and the corresponding calculated result is exhibited in Table S2. The *E_VB_* of R‐CeO_2_ and g‐C_3_N_4_ is estimated at +2.41 and +1.46 V (vs. NHE), respectively. Their *E_CB_* can be established as −0.54 and −1.36 V (vs. NHE) [46]. The specific bandgap structures of R‐CeO_2_ and g‐C_3_N_4_ are displayed in Figure S14a, which proves that R‐CeO_2_ has strong oxidation ability and g‐C_3_N_4_ has excellent reduction ability [57]. Based on the previous research, the VB of g‐C_3_N_4_ is not positive enough for the holes to oxidize the H_2_O to form hydroxyl radical (·OH, +1.46 V < +2.38 V vs. NHE), while the VB of R‐CeO_2_ has sufficient oxidation ability (+2.41 V > +2.38 V vs. NHE). At the same time, the CB of both materials is sufficiently negative for the electrons on CB to be able to react with O_2_ and obtain superoxide radical (·O_2_^−^, −0.54/−1.36 V< −0.33 V vs. NHE, as the Figure S14b) [58]. Therefore, the electron transport path at the interface can be determined according to the formation of radicals on the surface of the composite under the light irradiation. In situ electron spin resonance (ESR) spectra with DMPO as the spin capture agent were carried out to detect the superoxide (─O_2_^−^) and hydroxyl (─OH) radicals to further confirm whether the photogenerated electrons transfer path inside the CAC‐2 sample follows the S‐scheme path, and the corresponding result is displayed in Figure 7a,b. There are no DMPO‐O_2_^−^ and DMPO‐OH radical peaks before the light irradiation. Then, once the samples are exposed to the Xe‐lamp, the characteristic peaks of DMPO‐O_2_^−^ and DMPO‐OH radicals can be found. The in situ ESR spectra of DMPO‐O_2_^−^ and DMPO‐OH radicals’ curves were tested every 10 min, and their peak intensities gradually increased with the increase of light exposure‐time. The simultaneous enhancement of DMPO‐O_2_^−^ and DMPO‐OH radicals implies that the CAC‐2 ternary composite exhibits strong oxidation and reduction ability, which is fully consistent with the features of the S‐scheme heterojunction [59].

The surface characterization and CO_2_ adsorption ability of the prepared samples were tested by N_2_/CO_2_ absorption/desorption curves and DFT calculation. Figure S15a displays the N_2_ absorption/desorption curves of pure R‐CeO_2_, CC‐2, and CAC‐2, respectively. Their specific surface area is about 56.94, 74.79, and 116.08 cm^3^/g, respectively. The coupling of g‐C_3_N_4_ or Au can improve the specific surface area of the catalysts, which could efficiently improve the dispersion of the reduction sites (g‐C_3_N_4_). The CO_2_ initial adsorption amount (CO_2_ *IAA*) and net adsorption amount (CO_2_ *NAA*) values are obtained by the CO_2_ absorption/desorption curves in Figure S15b (Equation S2). At the same pressure, the CO_2_ *NAA* value of R‐CeO_2_, CC‐2, and CAC‐2 is about 1.09, 0.89, and 2.78 cm^3^/g, and the ternary composite exhibits greater CO_2_ adsorption performance than the other samples [60]. The variation trend of CO_2_ adsorption ability (CAC‐2> R‐CeO_2_> CC‐2) is inconsistent with that of their specific surface area (CAC‐2> CC‐2> R‐CeO_2_). Therefore, DFT calculation was used to compare the CO_2_ adsorption ability of the prepared materials, and the CO_2_ adsorption energy (*E_Ads_*) of the prepared catalysts was calculated [61].

The nanostructure and the corresponding *E_Ads_* of CeO_2_, CeO_2_/g‐C_3_N_4_, and CeO_2_/Au/g‐C_3_N_4_ are displayed in Figure 7c. Their *E_Ads_* is about −0.39, −0.11, and −0.74 eV, respectively. The CO_2_ adsorption ability decreases as follows: CAC‐2> R‐CeO_2_> CC‐2, which is consistent with the variation trend of CO_2_ adsorption ability tested by CO_2_ absorption/desorption curves in Figure S15b. CC‐2 exhibits the lowest CO_2_ adsorption ability compared to the pure R‐CeO_2_ and CAC‐2, which is likely due to the electron density reduction of g‐C_3_N_4_ after the formation of the S‐scheme between g‐C_3_N_4_/R‐CeO_2_. For the ternary composite, the introduction of Au NPs at the S‐scheme heterojunction interface effectively increases the electron density of the composite and maximizes the CO_2_ adsorption performance. In Figure 7d, g‐C_3_N_4_‐CO_2_, g‐C_3_N_4_/R‐CeO_2_‐CO_2_, and g‐C_3_N_4_/Au/R‐CeO_2_‐CO_2_ nanostructures have been built to compare the electron transfer efficiency between the catalyst and CO_2_ by Bader charge analysis. The net electron transfer from the catalyst to the CO_2_ molecule after the software optimization for the three structures is about 1.91, 1.95, and 2.08, respectively. In general, increased electron transfer means higher CO_2_ activation ability, and this increasing trend is consistent with the change of CO_2_ photoreduction performance of samples in Figure 4a (CAC‐2> CC‐2> g‐C_3_N_4_) [62]. The improved dispersion and electron density of the reduction sites are helpful for the CO_2_ photoreduction.

To analyze the CO_2_ photoreduction mechanism, the Gibbs free energies required for the formation of each important intermediate obtained by in situ FTIR were taken, and the corresponding results are exhibited in Figure 7e, and the specific nano‐structures of each step are shown in Figure S16. In this process, *CO_2_ is regarded as the initial state (0 eV), and the Gibbs free energy required for the formation of *COOH and *CO over different catalysts is shown in Table S6 [63]. The rate‐limiting step for CO_2_ photoreduction over CAC‐2 is the formation of the *COOH intermediate. The *COOH evolution barrier over CAC‐2 is the smallest compared with other samples, which is one of the main factors why CAC‐2 exhibits the excellent photoconversion efficiency of CO_2_ to CO [64, 65, 66].

### 2.7 CO_2_ Photoreduction Processes Analysis

**FIGURE 8:**
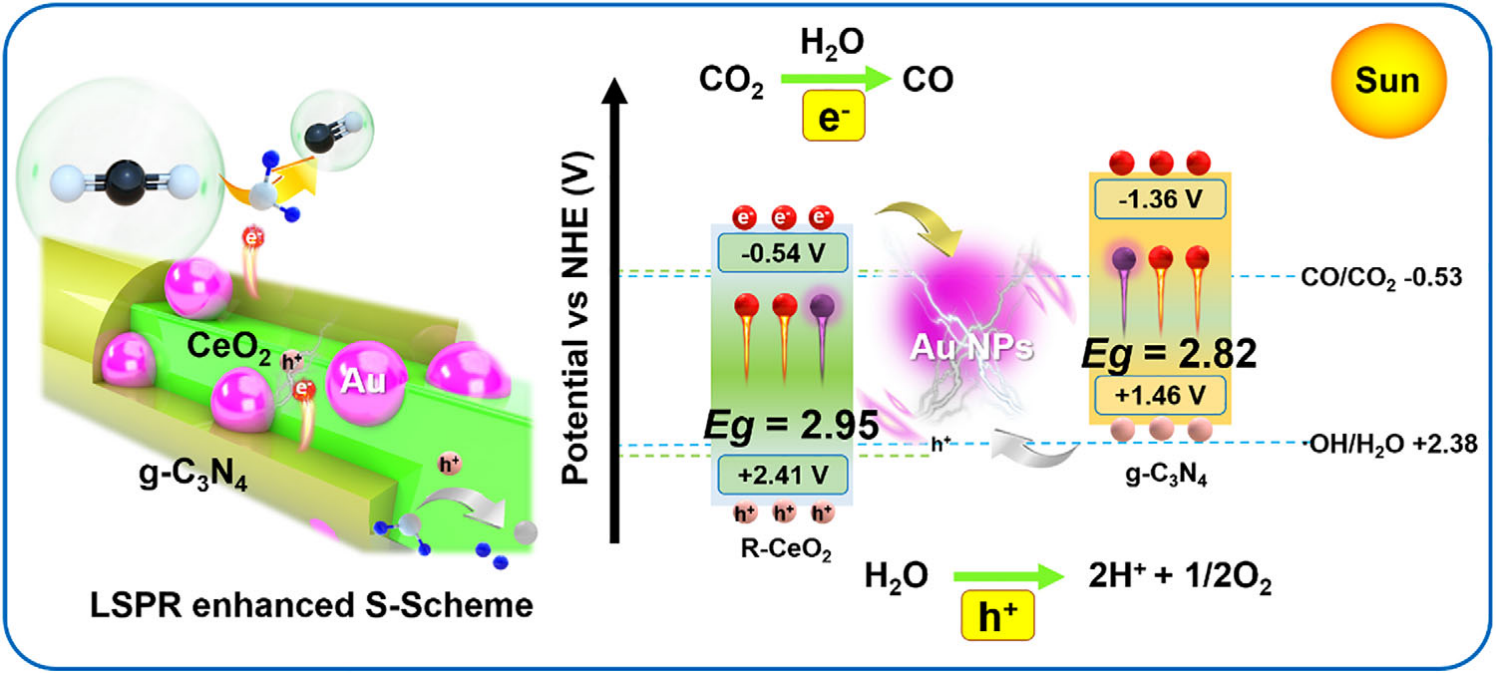
Au LSPR effect enhanced R‐CeO_2_/g‐C_3_N_4_ S‐scheme heterojunction for CO_2_ photoreduction under UV–vis light irradiation.

Based on the FDTD simulation, PEC tests, in situ FTIR, in situ ESR, Gibbs free energy calculation results, and our previous research, the potential enhanced CO_2_ photoreduction mechanism over Au LSPR modified CeO_2_/g‐C_3_N_4_ S‐scheme heterojunction has been provided in Figure 8 [67, 68]. First, a part of CO_2_ reacts with H_2_O to obtain the H_2_CO_3_, HCO_3_^−^, CO_3_^2−^ and H^+^ (Step 1), and another part of CO_2_ can be adsorbed on CAC‐2 (Step 2). Next, CeO_2_/g‐C_3_N_4_ and Au NPs can be excited by the UV–vis light, and the electrons photogenerated on the VB of CeO_2_ and g‐C_3_N_4_ can jump to the CB of them and the photogenerated holes left on the VB of them. Then, the electrons photogenerated on the CB of CeO_2_ recombine with the holes photogenerated on the VB of g‐C_3_N_4_ under the force of IEF at the binary interface, thus improving the separation efficiency of the electrons on the CB of g‐C_3_N_4_ and holes on the VB of CeO_2_ (Step3). At the same time, the LSPR effect of Au at the interface can greatly speed up the transmission and separation rates of the photogenerated carriers (Step 4). The H_2_O molecules can react with the photogenerated holes on the VB of CeO_2_ to form H^+^ and O_2_ (Step 5). Next, the separated electron on the CB of g‐C_3_N_4_ can directly react with CO_2_ attached on the surface of CAC‐2, and H^+^ to form the *COOH group (Step 6). Then, the obtained *COOH group further reacts with electrons and H^+^ to form *CO and H_2_O (Step 7). Finally, the free CO gas can be generated by a simple desorption process (Step 8) [69].

## 3 Conclusion

In this paper, a solid Au LSPR enhanced CeO_2_/Au/g‐C_3_N_4_ S‐scheme heterojunction has been successfully prepared and tested for the efficient CO_2_ photoreduction. The photocatalytic CO_2_ reduction experiments without a co‐catalyst and sacrificial agent show that the CAC‐2 exhibits the highest CO_2_ photoreduction performance compared to the pure samples and binary composites, and it has good catalytic stability. The CO yield on CAC‐2 is about 50.58 µmol·g^−1^·h^−1^ under the irradiation of UV–vis light, which is about 6.7 and 6.0 times higher than that of R‐CeO_2_ and g‐C_3_N_4_, respectively. Combining the experimental results and theoretical researches, we can find that the synergistic effect of R‐CeO_2_/g‐C_3_N_4_ S‐scheme heterojunction and the unique LSPR effect of Au NPs together maximize the photo‐absorption ability, separation of photogenerated carriers of the ternary composite and decrease the energy barrier of the formation of the important intermediate *COOH, thus obtaining the excellent CO_2_ photoreduction performance and 100% selectivity of CO. Besides, the improved dispersion and electron density of the reduction sites may be another reason for the enhancement of CO_2_ photoreduction ability. This paper not only offers a novel, green, and environmentally friendly catalyst for the photocatalytic CO_2_ reduction, but also provides some experimental and theoretical guidance for the construction of LSRP effect enhanced S‐scheme heterojunction catalytic system in the field of photocatalysis.

## 4 Experimental Section

### 4.1 Materials

In this paper, the cerium nitrate hexahydrate (Ce(NO_3_)_3_·6H_2_O), Sodium hydroxide (NaOH), Chloroauric acid (HAuCl_4_), and melamine were ordered from Sinopharm Group Co., Ltd. The CO_2_ gas (99.999%) was obtained from the Changchun Juyang Gas Co., LTD. 5,5‐Dimethyl‐1‐pyrroline N‐oxide (DMPO) and ^13^CO_2_ gas (99%) were bought from Sigma‐Aldrich.

### 4.2 Preparation of Photocatalysts

#### 4.2.1 Preparation of R‐CeO_2_

The R‐CeO_2_ was synthesized by a simple hydrothermal method followed by calcination. Typically, a certain amount of Ce(NO_3_)_3_·6H_2_O and NaOH was dissolved in about 50 mL of deionized water. The obtained mixture was stirred for about 15 min. Next, the solution was transferred into an 80 mL autoclave and heated to 120°C for 24 h. Then, the sediment was washed and dried for further use. Finally, the O_Vs_‐rich R‐CeO_2_ can be prepared after the calcination of the above sediment in air at about 500°C for 2 h.

#### 4.2.2 Preparation of R‐CeO_2_/Au

In this process, the ultraviolet reduction technology was applied to prepare the Au NPs enhanced R‐CeO_2_. 100 mg R‐CeO_2_ was added to 40 mL of deionized water and stirred for about 15 min. Then, a certain amount of HAuCl_4_ *·*4H_2_O aqueous solution (1, 3, 5, and 7 mL; concentration: 1 g/1000 mL) was added to the above mixture, and the suspensions were dispersed under UV‐light (λ≤ 280 nm) irradiation for about 0.5 h. After that, the obtained products were centrifuged, washed, and dried for 6 h. The obtained samples are labeled as CA‐1, CA‐2, CA‐3, and CA‐4, respectively.

#### 4.2.3 Preparation of G‐C_3_N_4_ Covered R‐CeO_2_/Au

**SCHEME 1:**
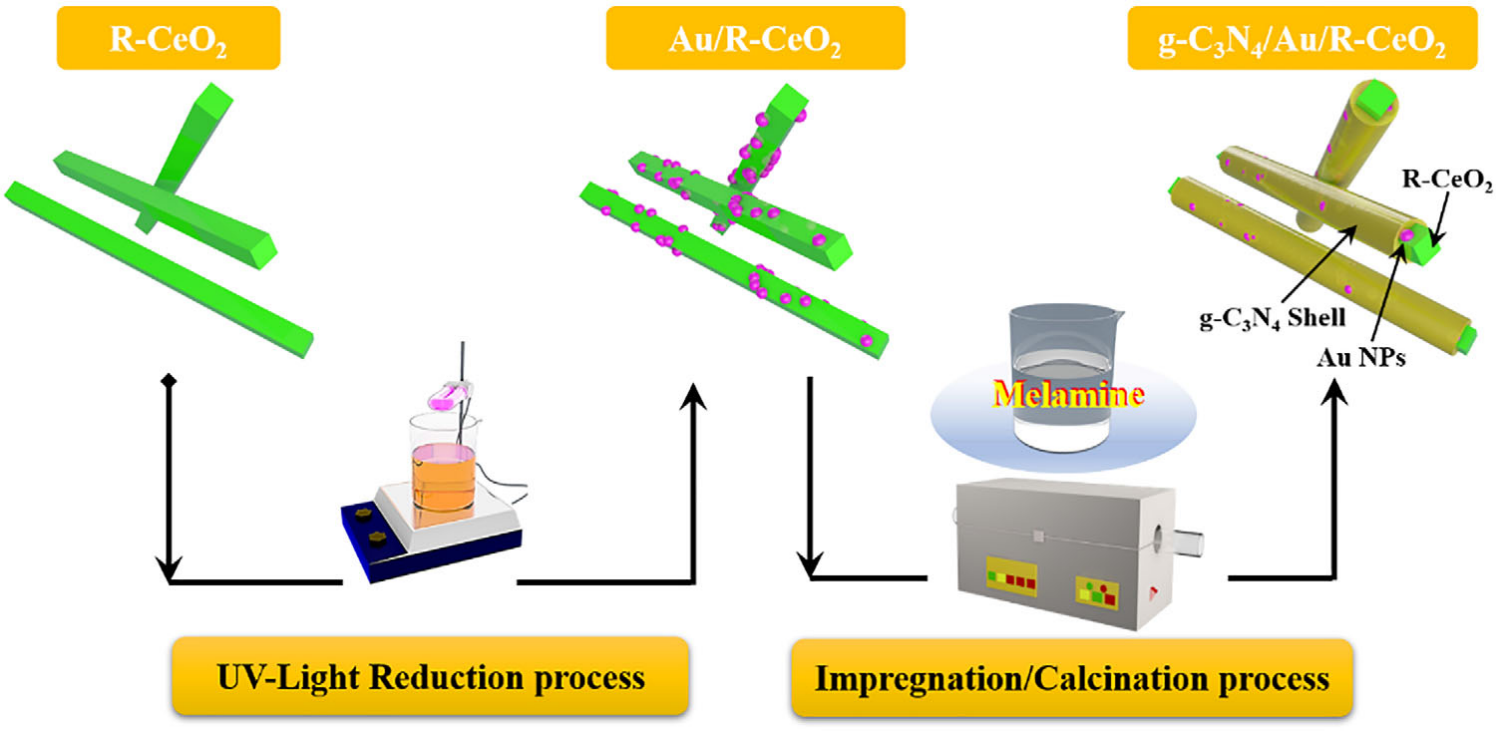
Synthesis steps of the catalysts.

For covering the g‐C_3_N_4_ on R‐CeO_2_/Au composite surface, the typical impregnation‐calcination method was carried out to synthesize the ternary composites. The precursor adsorption process: 100 mg R‐CeO_2_/Au (Optimum proportion, CA‐3) was dispersed into 40 mL saturated melamine methanol solution (60°C) and the obtained suspension was stirred for about 5 min to achieve the adsorption equilibrium between the precursor of g‐C_3_N_4_ (melamine) and R‐CeO_2_/Au. Finally, the g‐C_3_N_4_ shell covered Au/R‐CeO_2_ products were obtained after the calcination process in air (500°C, 2 h), and named as CAC‐1. To investigate the effect of g‐C_3_N_4_ shell loading amount, the ternary composites with different contents were adjusted by changing the precursor adsorption times, and named as CAC‐2, CAC‐3, and CAC‐4, respectively. Besides, for the purpose of facilitating comparison, we prepared the g‐C_3_N_4_‐coated R‐CeO_2_ complex with the same g‐C_3_N_4_ composite ratio as the CAC series, and the names of the obtained binary composites were labeled as CC‐1, CC‐2, CC‐3, and CC‐4. The corresponding preparation steps of the samples are shown in Scheme 1.

## Funding

National Natural Science Foundation of China (No. 22306142); the development of Science and Technology of Jilin province (YDZJ202301ZYTS246); The program for the Science and Technology of Education Department of Jilin Province (Grant No. JJKH20250941KJ); the open research topic of Key Laboratory of Functional Materials Physics and Chemistry, Ministry of Education (202403).

## Conflicts of Interest

The authors declare no conflicts of interest.

## Supporting information




**Supporting File**: smll72473‐sup‐0001‐SuppMat.docx.

## Data Availability

The data that support the findings of this study are available from the corresponding author upon reasonable request.
