# Supplementary material for: Au LSPR Effect Enhanced R‐CeO2/G‐C3N4 S‐scheme Heterojunction for Accelerating CO2 Photoreduction Performance
Source: Small. 2026 Jan 20;22(16):e12107. doi: 10.1002/smll.202512107 (PMC12994545; doi:10.1002/smll.202512107)
Supplement: Supplementary file 1 — Supporting File: smll72473‐sup‐0001‐SuppMat.docx. [file SMLL-22-e12107-s001.docx]

Supporting Information of

**Au LSPR effect enhanced R-CeO_2_/g-C_3_N_4_ S-scheme heterojunction for accelerating CO_2_ photoreduction performance**

Xin Li^a,b,†^, Yongsheng Hu^a,†^, Peng Tian^a^, Yi Lu^c^, Qiong Wu^a,b^, Binrong Li^d,^, Maobin Wei^a,b,^^[[1]](#footnote-1)^, Xiaofei Yang^c,*^, Lili Yang^a,b*^, Huilian Liu^a,b^, Alberto Vomiero^e,f*^

*^a^ Key Laboratory of Functional Materials Physics and Chemistry* *(Ministry of Education), Jilin Normal University, Changchun, 130103, China*

*^b^ National Demonstration Center for Experimental Physics Education, Jilin Normal University, Siping, 136000, China*

*^c^ International Innovation Center for Forest Chemicals and Materials, College of Science, Nanjing Forestry University, Nanjing, 210037 China*

*^d^ National and Local Joint Engineering Laboratory of Municipal Sewage Resource Utilization Technology, School of Environmental Science and Engineering, Suzhou University of Science and Technology, Suzhou 215009, PR China*

*^e^ Division of Materials Science, Department of Engineering Sciences and Mathematics, Luleå University of Technology, Luleå, 97187, Sweden*

*^f^ Department of Molecular Sciences and Nanosystems, Ca’ Foscari University of Venice, Via Torino 155, 30172 Venezia, Italy*

**1. Characterization.**

X-ray diffraction patterns (XRD) of powder samples were collected using a D/max2500PC rotating anode X-ray diffractometer with Cu Ked and point detectors in Bragg-Brentano geometry. The voltage was set to 40 kV, the current was set to 200 mA, and the sweep rate was 5º/min. The morphology and structure of the samples were characterized by transmission electron microscopy (TEM, JEOL JEM-HR2100). The surface morphology and element distribution of the samples were characterized by field emission scanning electron microscopy (SEM, JEOL, 7800F). The elemental composition of the sample surface was tested by X-ray photoelectron spectroscopy (XPS) (Esca Lab 250 XI). The peak position of C 1s was set to 284.6 eV. PL spectra were obtained by an Edinburgh-Steady State/Transient Fluorescence Spectrometer FLS1000 (Edinburgh Instrument Company, UK). The UV-Vis absorption spectrum was measured with a Shimadzu UV-3600Plus spectrophotometer and the test range is from 200 nm to 800 nm. The photoelectrochemical properties of samples, using transient photocurrent response (TPR), electrochemical impedance spectroscopy (EIS), linear sweep voltammetry (LSV), and Mott-Schottky curves were studied on an electrochemical workstation (CHI 660B, Shanghai, China). The standard three-electrode quartz model with an Ag/AgCl electrode has been used in the test processes. A solution with NaSO_4_ was used in the EIS test process. Specific surface area and N_2_ adsorption-desorption isotherm curve were determined by gas adsorption apparatus (BSD 660, BEISHIDE instrument tec. Co LTD). CO_2_ and CO desorption curves were determined by fully automated chemisorption apparatus (BSD-Chem C200, BEISHIDE instrument tec. Co LTD), respectively. The specific CO_2_ photoreduction process was recorded on the *in-situ* FTIR by the Nicolet iS50, USA. The *in-situ* FTIR was tested by the following process: Before the test, the catalyst was pretreated at 120°C for 2 h under vacuum to remove impurity molecules adsorbed on the catalyst surface, cooled and then 50 mg catalyst, 2 mL deionized water was added into the *in-situ* reactor. Then, the N_2_ was used to purge the impurity gas in the *in-situ* FTIR system. Next, the background was collected in the dark and the CO_2_ gas was slowly injected into the reaction system. And the *in-situ* FTIR began to record the spectrum every 2 min, and the dart test was completed after 14 min. After that, the background signal was collected again and the baseline was calibrated. The 10 W UV LED-lamp (365 nm) and 300W Xe-lamp were turned on to start the *in-situ* FTIR test of CO_2_ photoreduction reaction with CAC-2 as the catalyst under the Xe-light irradiation and the FTIR curve was collected after each 5 min, and the reaction lasted for 35 minutes. The ^13^CO_2_ isotope test was carried out by replacing the CO_2_ gas with a mixture of Ar gas and ^13^CO_2_ under the same reaction conditions as the CO_2_ photoreduction process, and the final product was analyzed by gas chromatography-mass spectrometry (GC-MS, Agilent USA, 7890B-5977B). The EPR/*in-situ* ESR results were detected by a Bruker electron paramagnetic resonance spectrometer (Micro ESR, Bruker Corporation, Germany). In the *in-situ* ESR test process, 5,5-Dimethyl-1-pyrroline N-oxide (DMPO) was used as the spin capture agent and a 300 W Xe-lamp was applied as the light source. The ESR spectra were repeated 4 times under the same illumination conditions (after each 10 min). Thus, three-dimensional data of field strength-ESR signal strength-light exposure time can be obtained.

**2. CO_2_ photoreduction**

The photocatalytic CO_2_ reduction activity of the prepared photocatalysts was determined using a closed 300 mL photo-chemical reactor under the UV-Visible light irradiation provided by the Xe-lamp (PLS-SXE300D, 300 W, Beijing Perfectlight Technology Co., LTD). In the photoreduction process, the pure CO_2_ (99.999%) was bubbled into the mixed liquid with 20 mg prepared composite, 50 mL water for 30 min to remove the impurities gases. The distance of the Xe-lamp to the liquid is 16 cm. The total optical power impinging on the mixture liquid is about 13.88 mW/mL, 320-780 nm. After irradiation for 4 h, the products were quantitatively analyzed by gas-chromatography (GC-8670M, Nanjing Dongcun Scientific Instrument Co., Ltd., China/GC-2014, Shimadzu, Japan). The electron utilization (electron flux, *R*) of our prepared samples were calculated by the formular:

*R* (electron) = 2*r*(CO) + 8*r*(CH_4_) (1)

where *r* is the formation efficiency of CO or CH_4_, respectively.

**3. DFT methods:**

Structural optimization and property calculations are performed within the density functional theory framework, as implemented in the Vienna ab initio simulation package (VASP), where the ion−electron interaction is implemented by the projector-augmented plane wave (PAW) approach. The structural models, volumetric data such as electron/nuclear densities, and crystal morphologies are processed by VESTA (Visualization for Electronic Structural Analysis). The electronic exchange-correlation functional is treated using the generalized gradient approximation (GGA) in the form proposed by Perdew, Burke, and Ernzerhof (PBE). The energy cutoff of the plane waves is set to 550 eV, with an energy precision of 10^−6^ eV. Atomic positions are fully relaxed until the force on each atom is less than 10^−3^ eV/Å. The supercell method is considered to simulate the monolayer, where a vacuum distance of ∼30 Å is used to eliminate the interaction between adjacent layers.

**Table S1.** The full XPS peak fitting information of Ce ions.

| **Peak** | **Binding Energy (eV)** | **FWHM** | **Peak area** | **at%** |
| --- | --- | --- | --- | --- |
| **1-Ce^3+^** | 882.5 | 2.18 | 133765.6 | 10.54 |
| **2-Ce^3+^** | 884.5 | 4.58 | 224874.4 | 17.72 |
| **3-Ce^3+^** | 889.1 | 4.63 | 192275.9 | 15.15 |
| **4-Ce^3+^** | 898.3 | 2.18 | 173402.4 | 13.67 |
| **1-Ce^4+^** | 901.1 | 2.80 | 147775.6 | 11.65 |
| **2-Ce^4+^** | 903.7 | 3.22 | 98316.8 | 7.75 |
| **3-Ce^3+^** | 907.3 | 3.22 | 112983.3 | 8.91 |
| **4-Ce^4+^** | 917.0 | 2.95 | 185349.2 | 14.61 |

Ce^3+^/Ce^4+^

=SCe^3+^/SCe^4+^

=S(1-Ce^3+^+2-Ce^3+^+3-Ce^3+^+4-Ce^3+^)/S(1-Ce^4+^+2-Ce^4+^+3-Ce^4+^+4-Ce^4+^) =(133765.6+224874.4+192275.9+173402.4)/(147775.6+98316.8+112983.3+185349.2)

≈1.33


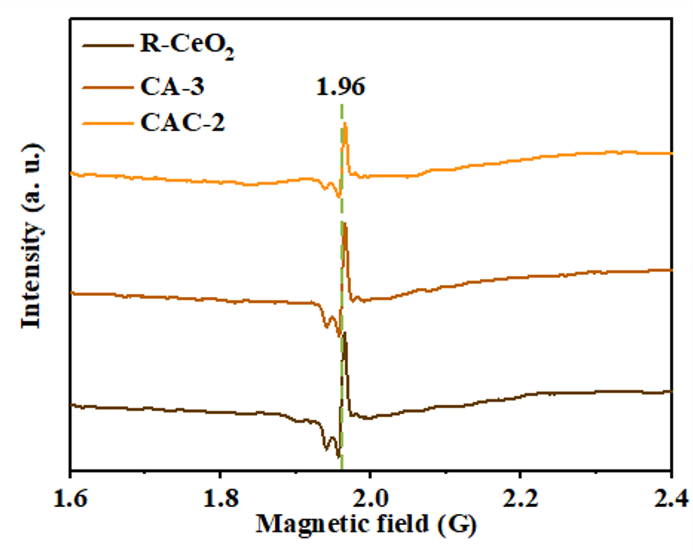


**Figure S1.** EPR curves of all samples.





**Figure S2.** EDS energy spectrum of CAC-2.

**Table S2.** The content of each element determined by EDS energy spectrum

| Element | C | N | Au | Ce | O |
| --- | --- | --- | --- | --- | --- |
| *wt*% | 31.6% | 12.8% | 3.5% | 41.8% | 10.3% |


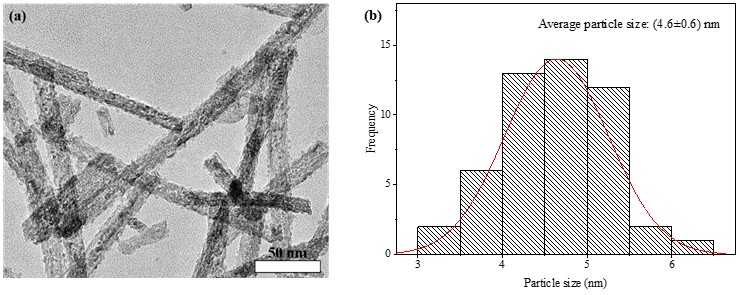


**Figure S3.** The TEM image of CAC-2 (a) and the corresponding Au NPs size histogram with Gaussian fit (red line) (b).


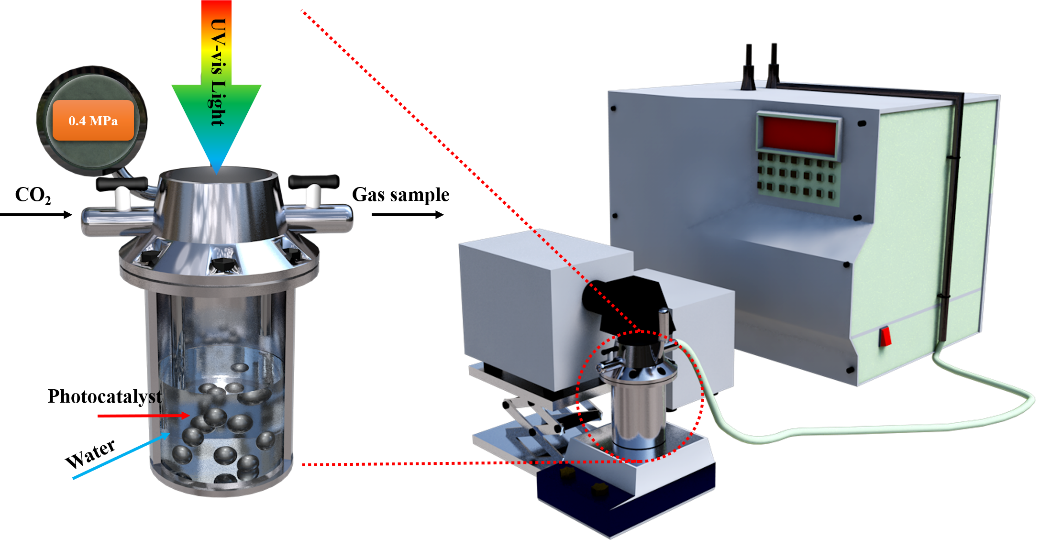


**Figure S4**. The specific CO_2_ photoreduction system.

**Table S3.** CO and CH_4_ yield of each catalyst

| Photocatalyst | CO Yield (µmol·g^-1^·h^-1^) | CH_4_ Yield (µmol·g^-1^·h^-1^) | CO Selectivity |
| --- | --- | --- | --- |
| R-CeO_2_ | 7.50 | 0.21 | 97.28% |
| CA-1 | 10.33 | 0.84 | 92.48% |
| CA-3 | 24.62 | 3.80 | 86.63% |
| CA-5 | 16.24 | 1.72 | 90.42% |
| CA-10 | 14.59 | 2.37 | 86.03% |
| CAC-1 | 42.14 | 0.00 | 100.00% |
| CAC-2 | 50.58 | 0.00 | 100.00% |
| CAC-3 | 33.66 | 0.00 | 100.00% |
| CAC-4 | 26.63 | 0.67 | 97.55% |
| CC-1 | 24.77 | 3.24 | 88.43% |
| CC-2 | 30.53 | 3.88 | 88.72% |
| CC-3 | 18.61 | 2.45 | 88.37% |
| CC-4 | 12.45 | 1.83 | 87.18% |
| CN | 8.38 | 1.50 | 84.82% |


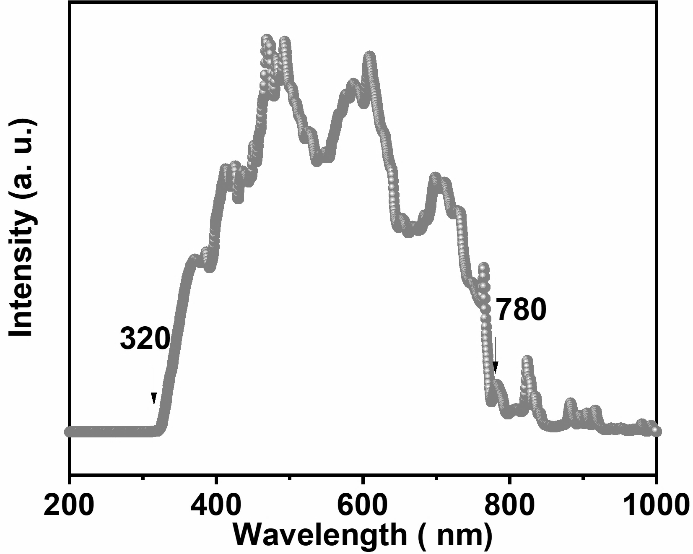


**Figure S5.** The specific Xe-lamp emission spectrum.





**Figure S6.** The CO yield with CC-2 and CAC-2 as the catalyst under the irradiation of Visible light.





**Figure S7.** N 1s and C 1s XPS spectra of CAC-2 before and after the CO_2_ photoreduction.





**Figure S8.** The CO, CH_4_, O_2_ yield of CAC-2 in CO_2_ PR reaction.

**Table S4.** The CO, CH_4_, O_2_ yield of CAC-2 in CO_2_ PR reaction.

| **Products** | **1 (µmol·g^-1^·h^-1^)** | **2 (µmol·g^-1^·h^-1^)** | **3 (µmol·g^-1^·h^-1^)** | **Average (µmol·g^-1^·h^-1^)** |
| --- | --- | --- | --- | --- |
| **CO** | 52.48 | 55.07 | 51.44 | 53.00 |
| **CH_4_** | 0.00 | 0.00 | 0.00 | 0.00 |
| **O_2_** | 21.46 | 22.08 | 18.17 | 20.57 |


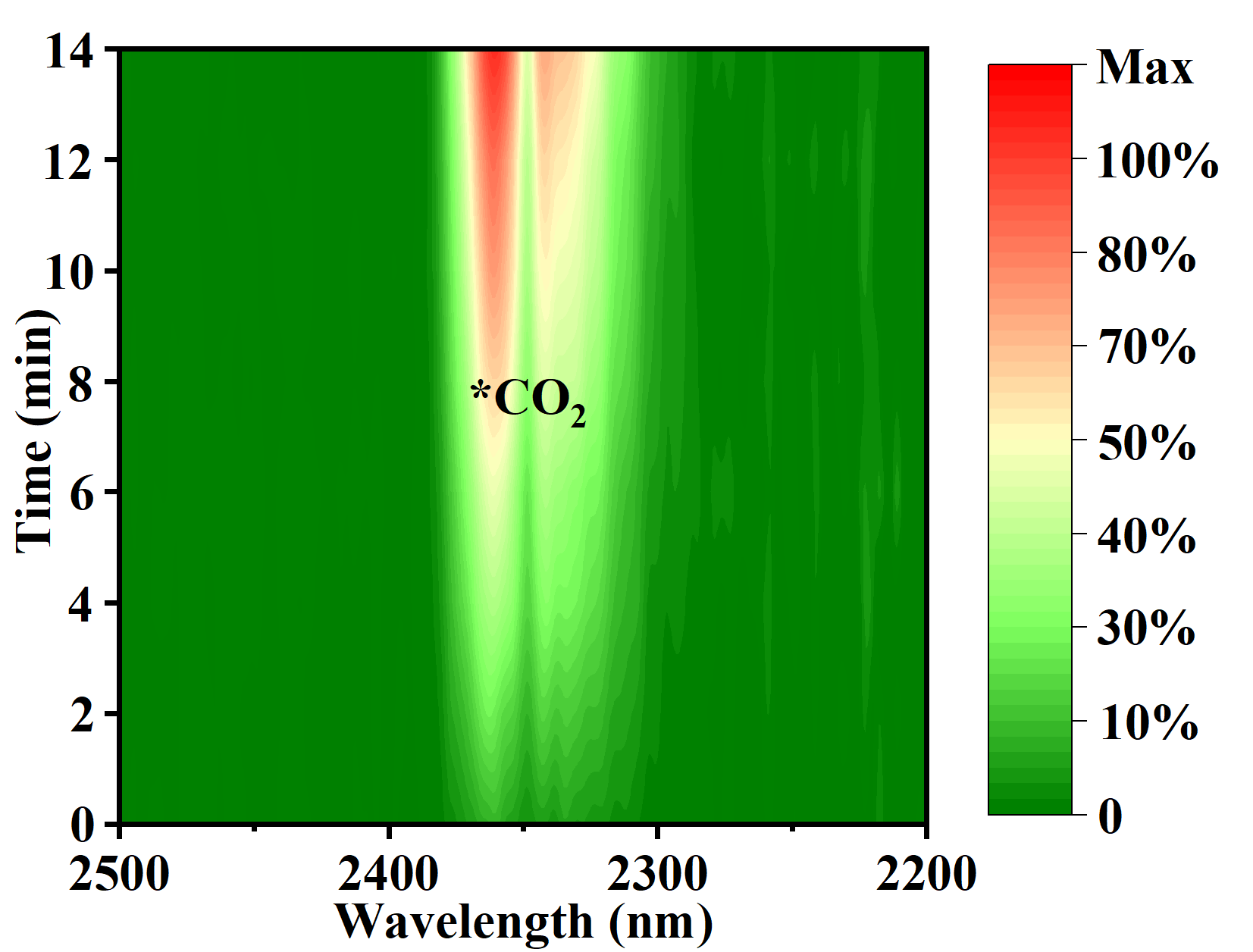


**Figure S9.** *In-situ* FTIR spectra of CO_2_ adsorption process on the surface of CAC-2.


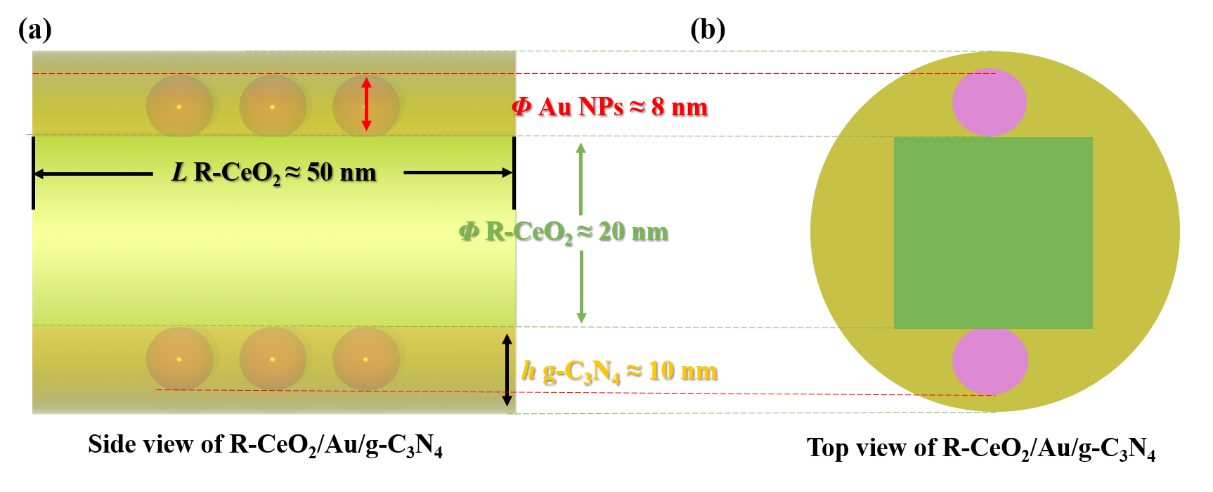


**Figure S10.** The R-CeO_2_/Au/g-C_3_N_4_ model used in FDTD simulations, side view (a) and top view (b).


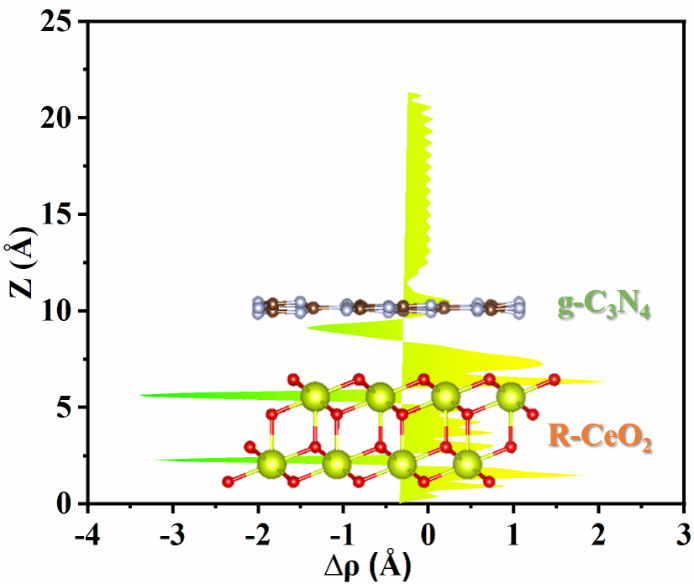


**Figure S11.** The averaged charge density (*Δρ*) as a function of the vertical axis integrating all the cyan and yellow regions of Figure 6g.


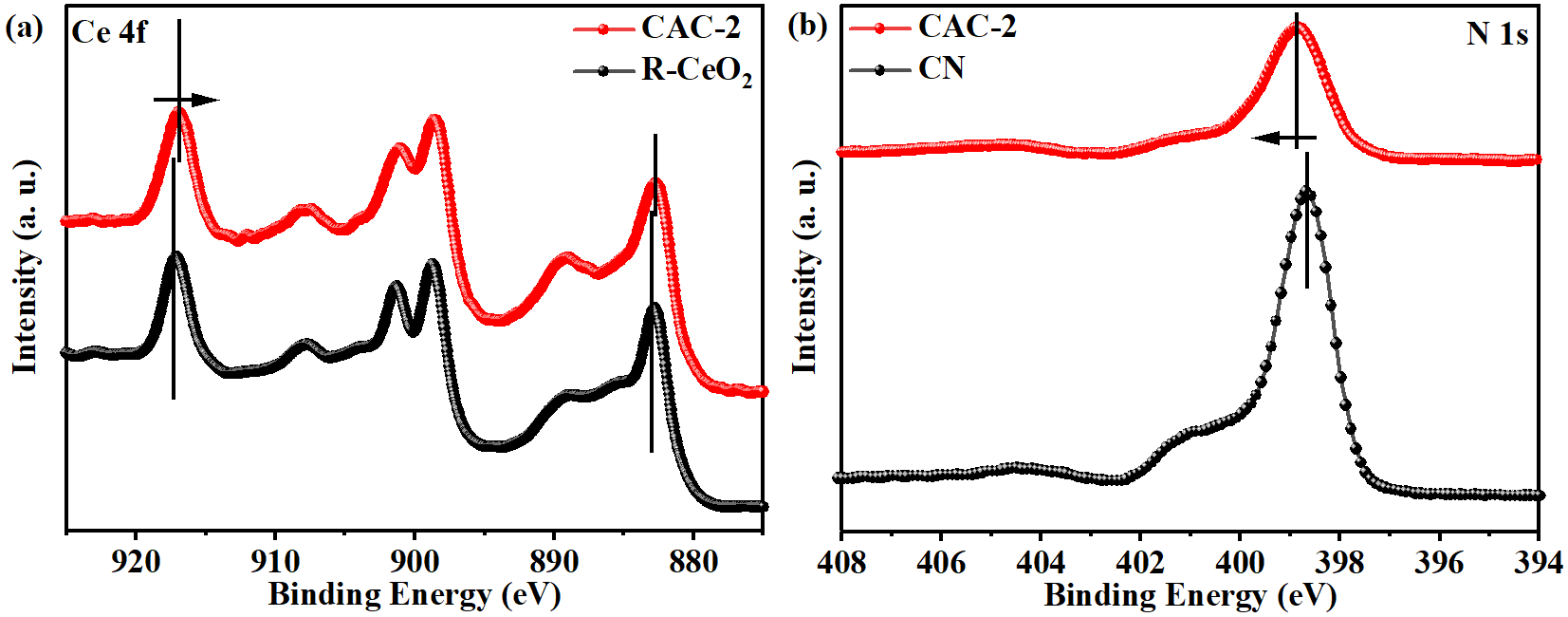


**Figure S12.** Ce 4f (a) and N 1s (b) XPS spectra of CAC-2, and pure R-CeO_2_ or CN.


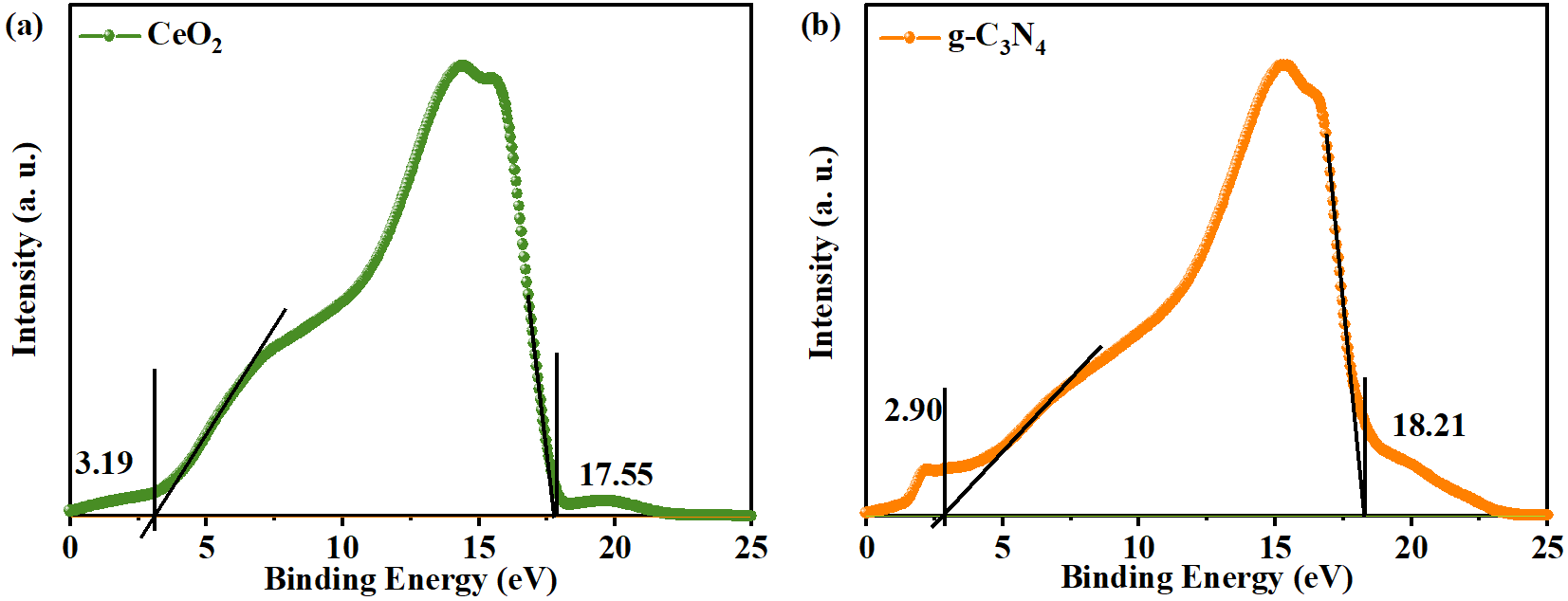


**Figure S13.** UPS spectra of R-CeO_2_ (a) and g-C_3_N_4_ (b).

**Tatal S5.** The specific calculation results for the band gap sturctrue.

| Sample | *hv* | *E_cut-off_* | *E_Fermi_* | *E_g_* | *Φ* | *E_VB_* | *E_CB_* |
| --- | --- | --- | --- | --- | --- | --- | --- |
| R-CeO_2_ | 21.22 | 17.55 | 3.19 | 2.95 | 3.67 | +2.41 | -0.54 |
| g-C_3_N_4_ | 21.22 | 18.21 | 2.90 | 2.82 | 3.01 | +1.46 | -1.36 |


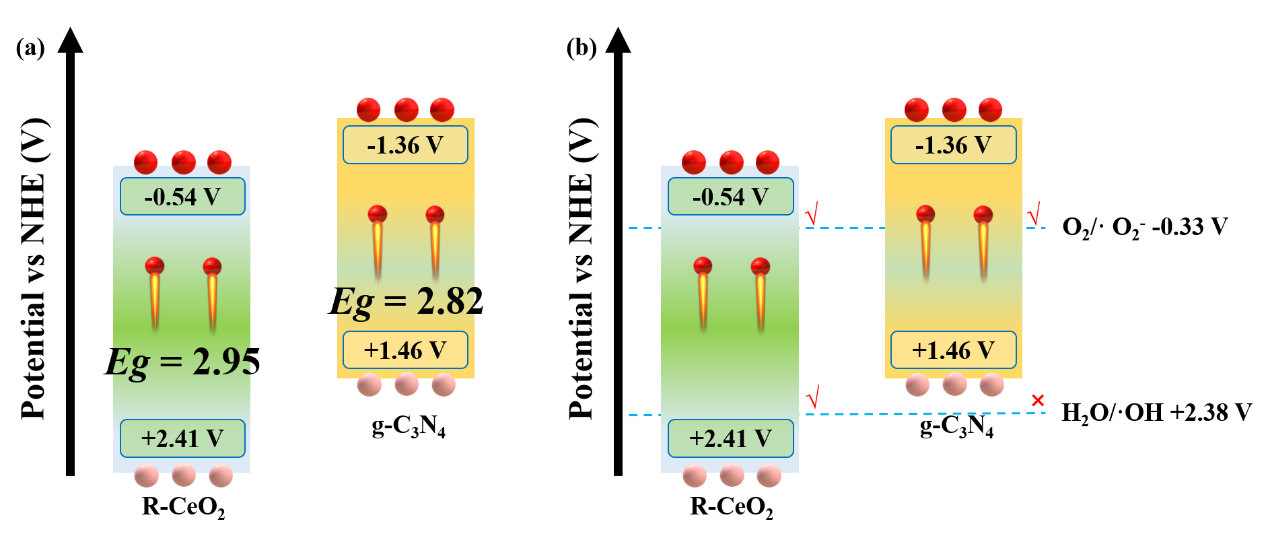


**Figure S14.** Band gap structure (a) and the radical generating ability (b) of R-CeO_2_ and g-C_3_N_4_.


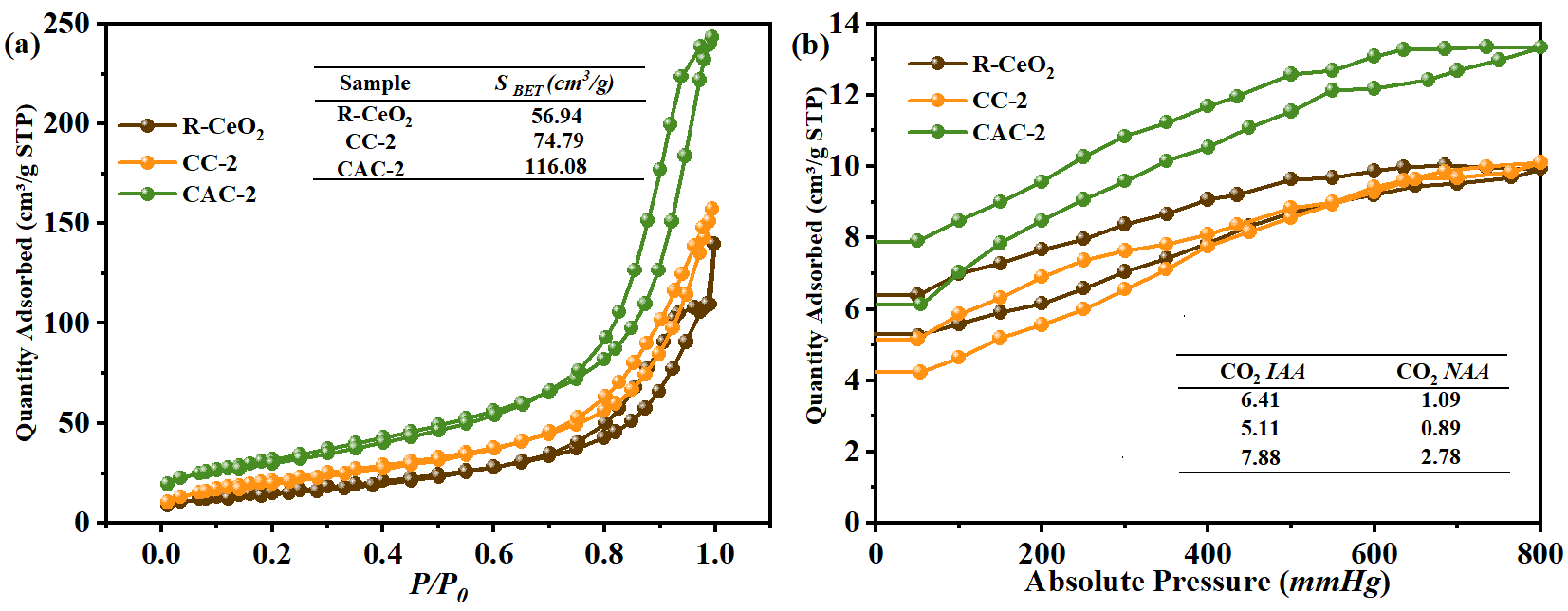


**Figure S15.** The BET and CO_2_ adsorption ability of prepared samples.

**CO_2_** **Initial Adsorption Amount (CO_2_ IAA):** Adsorption amount of CO_2_ at atmospheric pressure before the adsorption process.

**CO_2_** **Final Adsorption Amount of CO_2_ (CO_2_ FAA):** Adsorption amount of CO_2_ at atmospheric pressure after the desorption process.

**CO_2_ Net Adsorption Amount (CO_2_ *NAA*):** The amount of CO_2_ still adsorbed on the catalyst surface after the absorption and desorption process

CO_2_ *NAA =* CO_2_ *IAA* - CO_2_ *FAA* (S2)


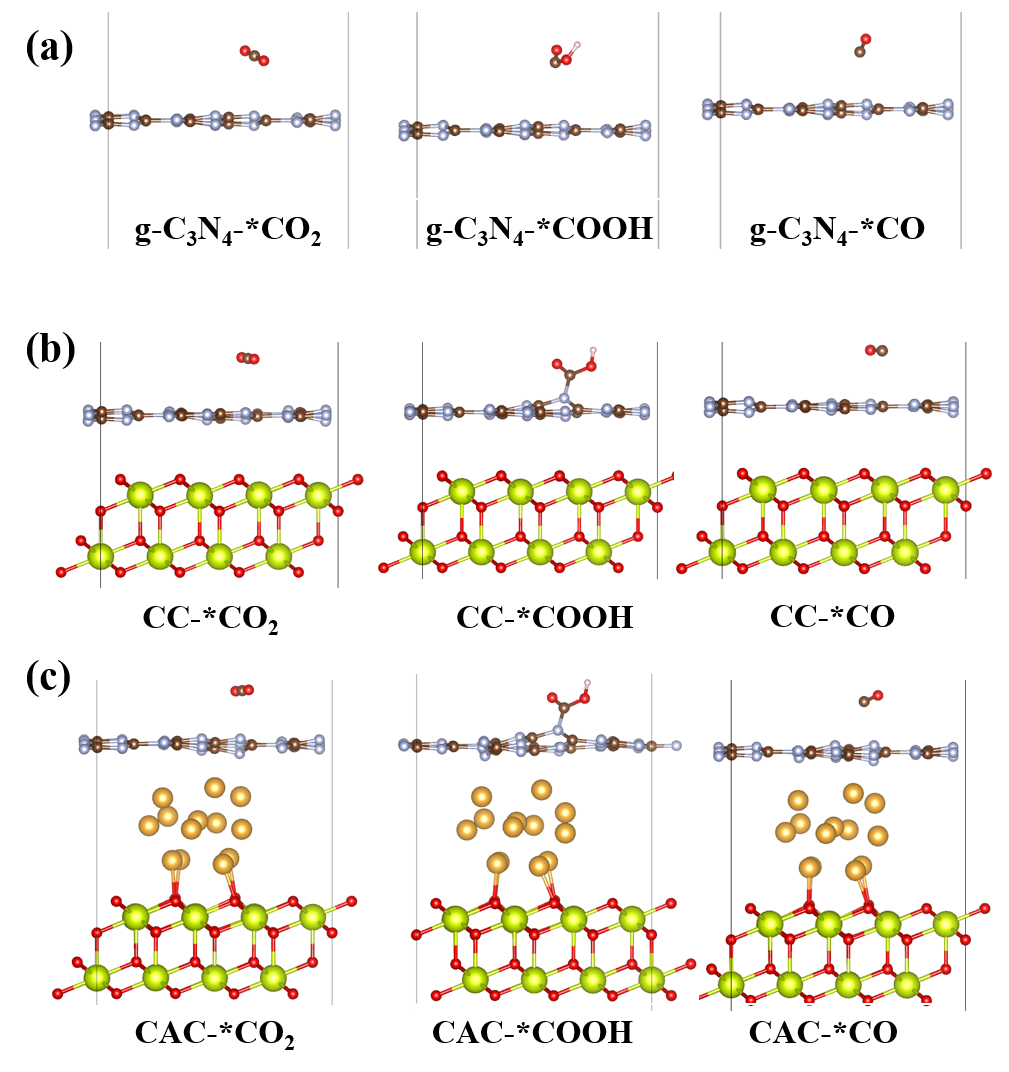


**Figure S16.** Nano-structures of each reaction coordinate on g-C_3_N_4_, g-C_3_N_4_/CeO_2_, and g-C_3_N_4_/Au/CeO.

**Table S6.** Gibbs free energy of each reaction coordinate on CeO_2_, CeO_2_/Au and CeO_2_/Au/g-C_3_N_4_.

| **Sample** | ***CO_2_** | ***COOH** | ***CO** |
| --- | --- | --- | --- |
| **CeO_2_** | 0.00 | +1.62 | +0.35 |
| **CeO_2_/Au** | 0.00 | +0.67 | +0.62 |
| **CeO_2_/Au/g-C_3_N_4_** | 0.00 | -0.46 | +0.07 |

1. † They have the same contribution to this paper.

   Corresponding authors E-mail addresses: jlsdzccw@126.com (Maobin Wei); xiaofei.yang@njfu.edu.cn (Xiaofei Yang); llyang1980@126.com (Lili Yang), alberto.vomiero@ltu.se (Alberto Vomiero). [↑](#footnote-ref-1)
